# Microbial oxidation significantly reduces methane export from global groundwaters

**DOI:** 10.1073/pnas.2508773122

**Published:** 2025-10-13

**Authors:** Beatrix M. Heinze, Valérie F. Schwab, Kirsten Küsel, Stefan Schloemer, Andreas Roskam, Xiaomei Xu, Susan Trumbore

**Affiliations:** ^a^Department Biogeochemical Processes, Max-Planck-Institute for Biogeochemistry, Jena 07745, Germany; ^b^Aquatic Geomicrobiology, Institute of Biodiversity, Ecology and Evolution, Friedrich Schiller University, Jena 07743, Germany; ^c^Cluster of Excellence Balance of the Microverse, Friedrich Schiller University Jena, Jena 07745, Germany; ^d^German Center for Integrative Biodiversity Research, Halle-Jena-Leipzig 04103, Germany; ^e^Federal Institute for Geosciences and Natural Resources, Hanover 30655, Germany; ^f^State Agency for Water Management, Coastal and Nature Conservation, Aurich 26603, Germany; ^g^Department of Earth System Science, University of California, Irvine, CA 92697

**Keywords:** microbial methane oxidation, groundwater, ^14^C-rate measurement

## Abstract

Methane is ubiquitous in groundwater, and its release to surface environments through pumping, discharge, or diffusion is an emerging environmental concern. Microbial oxidation consumes methane and mitigates its release, but quantitative constraints in groundwater remain unknown. Using ultra-low-level ^14^C-labeling, we estimate in situ microbial methane oxidation rates in shallow carbonate and sandy aquifers from central and northern Germany with methane concentrations spanning 5 orders of magnitude, from 0.15 ± 0.04 to 36,250 ± 1,390 µg L^−1^. Oxidation rates ranged from 0.001 ± 0.0003 to 74.28 ± 46.94 µgC L^−1^ d^−1^ and were highly correlated with groundwater methane concentrations. Oxidation-based methane turnover was rapid at low methane concentrations, with complete consumption requiring days to weeks. In contrast, microbial oxidation at high methane sites required months to decades for complete methane turnover, indicating the potential for unconsumed methane to leak into local streams or wetlands. High oxidation rates were associated with *gammaproteobacterial* methanotrophs that typically thrive in suboxic conditions and anaerobic methane-oxidizing archaea, while uncultivated methanotrophs of the *Methylomirabilota* and *Verrucomicrobiota* dominated low-rate sites. Based on globally distributed groundwater methane concentration data, we extrapolated the strong observed correlation between methane concentrations and oxidation rates to global groundwater volumes, estimating that microbial oxidation removes ~66% of groundwater methane globally, equivalent to 167 to 778 Tg CH_4_ y^−1^. This highlights the groundwater microbiome as a crucial subsurface methane filter that reduces methane release to surface waters, soils, and the atmosphere.

## Significance

Most groundwaters contain methane, produced either microbially or from fossil sources. At higher concentrations, methane poses a concern for drinking water quality and may escape to soils, surface waters, and the atmosphere, where it can contribute to global warming. However, freshwater contributions to global methane budgets are highly uncertain. This study quantifies microbial oxidation, the main methane sink, in groundwaters with different bedrock lithologies. It further links the functioning of this filter to methanotrophic communities and hydrogeochemical conditions. By extrapolating the relationships found, we provide a global-scale estimate of groundwater methane oxidation. This work establishes a critical baseline for future research on methane cycling in groundwater, an ecosystem increasingly affected by hydroclimatic extremes and anthropogenic activities.

Methane is a potent greenhouse gas, accounting for approximately 30% of current global warming (1), with aquatic ecosystems contributing more than half of natural methane emissions (2, 3). Groundwater, the largest terrestrial freshwater reservoir (4), contains dissolved methane across diverse bedrock lithologies (5–8), with concentrations reaching supersaturated levels (9, 10). Groundwater methane is released into surface environments through pumping for irrigation or drinking water supply (5, 11, 12), seepage into lakes and wetlands (13–16), baseflow into streams and rivers (17–20), or submarine groundwater discharge (21–23). Particularly in the Arctic, methane emissions from groundwater discharge are intensifying due to thawing permafrost and glacial meltwater runoff (10, 13, 24, 25). Hydrological events or anthropogenic activities may therefore rapidly release stored groundwater methane, increasing its contribution to the atmospheric methane budget. Microbial oxidation is the only biological methane sink, removing up to 90% of the methane released from cold seeps (26), shallow marine environments (27), wetlands (28), and lakes (29, 30). However, while methane oxidation is well characterized in these systems, quantitative constraints on rates of microbial methane consumption in global aquifers remain unknown, limiting our ability to assess the role of freshwater ecosystems in the global methane budget.

Groundwater methane originates primarily from microbial methanogenesis (31, 32) and thermogenic sources introduced via hydraulic fracturing (33, 34) or upward gas migration (35–37). High groundwater methane concentrations are often linked to biogenic production (8, 32, 38–40), though elevated levels of thermogenic methane have been detected near natural gas extraction sites (41, 42). Biogenic methane, produced mainly by archaea under anoxic conditions (43, 44), is typically ^13^C-depleted compared to thermogenic methane, which originates from the thermal decomposition of buried organic matter (45). Additional indicators of thermogenic gas are the absence of the radioactive carbon-14 isotope (^14^C-free methane due to radioactive decay over the millions of years since its carbon was originally fixed from the atmosphere) (46) and the presence of ethane or propane (47).

Microbial methane oxidation occurs under both oxic and anoxic conditions. Aerobic methane oxidation (MOx) is mediated by methanotrophs from the *Gammaproteobacteria* (Type I)*, Alphaproteobacteria* (Type II), and *Verrucomicrobiota* (Type III), which utilize particulate (Pmo) or soluble (Mmo) methane monooxygenase for methane activation (48, 49). Anaerobic methane oxidation (AOM), coupled to sulfate, nitrate, nitrite, iron- or manganese reduction, is performed by members of the *Methylomirabilota*, and the archaeal *Halobacteriota* (48), including freshwater-associated *Methanoperedenaceae* (50). While bacterial anaerobic methanotrophs generate oxygen intracellularly to oxidize methane (51), archaeal anaerobic methane oxidizers (ANME) use the reverse pathway of archaeal methane production (52). Methanotrophs are widespread in groundwater (53–55), sometimes comprising over 40% of the microbial community (56). However, their abundance and activity depend on methane availability and environmental conditions, which in turn regulate oxidation rates (57–59).

We quantified in situ microbial methane oxidation rates in carbonate and sandy aquifers from central and northern Germany by adding ultra-low levels of ^14^C-labeled methane. We assessed ^14^C-CH_4_ oxidation rates from the conversion of the ^14^C-label to dissolved inorganic carbon (DIC) and methane assimilation rates from ^14^C-label uptake into particulate organic matter (POM). Rate measurements were combined with marker gene quantification, 16S rRNA sequencing, and methane isotope analysis to i) determine the range and variability of microbial methane oxidation rates in groundwater, ii) identify key methanotrophic taxa associated with high- and low-rate sites, and iii) evaluate environmental constraints on groundwater methane turnover. Our findings provide quantitative boundaries for microbial methane removal in groundwater and offer a first-order estimate of the global significance of groundwater methane oxidation as a subsurface methane filter.

## Results

### Groundwater Methane Concentrations, Oxidation Rates, and Turnover Times.

**Fig. 1.**
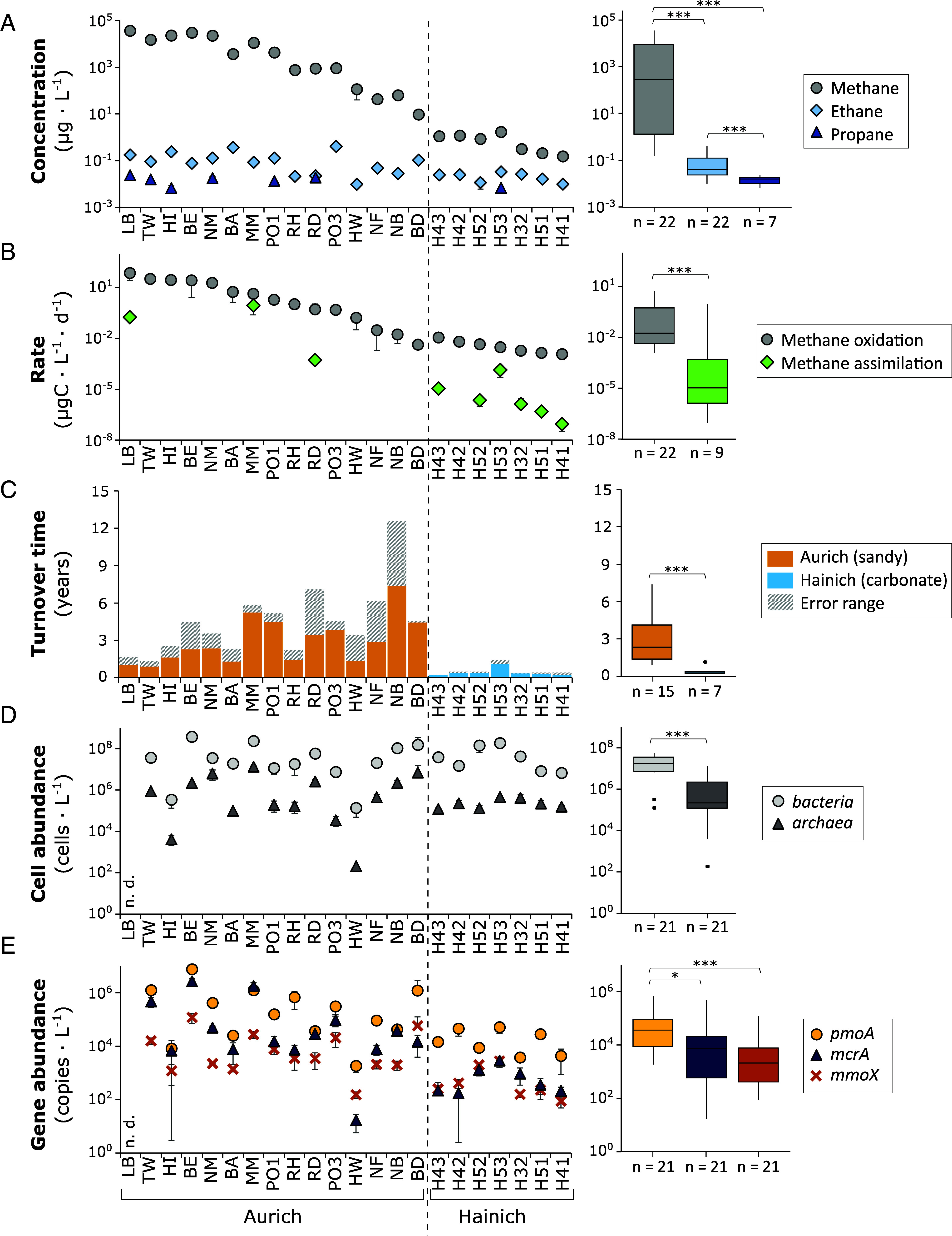
Rates, turnover times, and gene abundances across the aquifers. (*A*) Groundwater methane (gray), ethane (light blue), and propane (dark blue) concentrations. Error bars represent SD between 2 (Aurich) or 3 (Hainich) replicate samples. (*B*) Rates of methane oxidation (gray) and methane carbon fixation (green). Error bars represent SD of Monte Carlo error propagation. (*C*) Methane turnover times calculated from methane oxidation rates and methane concentrations. Errors from rate and concentration measurements were combined using Taylor series expansion. (*D*) Bacterial and archaeal cell abundances derived from quantitative PCR of the 16S rRNA gene corrected for gene copy numbers. Error bars represent SD between three replicate samples. (*E*) Abundance of methane oxidation genes per L groundwater derived from quantitative PCR. Error bars represent SD between three replicate samples. *pmoA*: Particulate methane monooxygenase. *mcrA*: Methyl-coenzyme M reductase. *mmoX*: Soluble methane monooxygenase. Wilcoxon rank-sum test: *P < 0.05 **P < 0.01, and ***P < 0.001.

Groundwater methane concentrations ranged over five orders of magnitude, from 1.5 · 10^−4^ ± 4.0 · 10^−5^ to 36.25 ± 1.39 mg L^−1^ (mean ± stdev, n = 22, Fig. 1*A*). Consistent with previous measurements, the Hainich carbonate groundwater had very low methane concentrations (60), while higher concentrations were found in porous sandy aquifers around Aurich (8). Methane oxidation rates were lowest at the carbonate site, ranging from 0.001 ± 0.0003 to 0.01 ± 0.001 µgC L^−1^ d^−1^ (0.0001 to 0.0009 µmol CH_4_ L^−1^ d^−1^), but as high as 74.28 ± 46.94 µgC L^−1^ d^−1^ (6.18 µmol CH_4_ L^−1^ d^−1^) in the porous sand setting (mean ± stdev, Monte Carlo error propagation, Fig. 1*B* and Dataset S1 *A*–*F*). Groundwater methane oxidation rates and methane concentrations were highly correlated (r = 0.9650, *P* = 4.3 · 10^−13^, n = 22, Spearman correlation, *SI Appendix*, Figs. S2 and S3 and Dataset S4 *A*–*C*).

Methane turnover times, i.e. the time required to oxidize the in situ methane pool, differed between the two groundwater settings (Fig. 1*C*). Methane turnover times in the Hainich wells (n = 7), ranging from 0.2 ± 0.04 to 1.1 ± 0.3 y, averaged lower than the Aurich site (n = 15), ranging from 0.9 ± 0.4 to 7.4 ± 5.2 y (W = 103, *P* = 4.7 · 10^−5^, Wilcoxon rank-sum test). Methane turnover times correlated with methane concentration (r = 0.5133, *P* = 0.02, Spearman correlation, *SI Appendix*, Fig. S3), but less strongly than methane oxidation rates.

### Methanotroph Abundance and Cell-Specific Methane Oxidation Rates.

Total microbial cell numbers were similar between groundwater of the sandy and carbonate aquifers, ranging from 1.34 · 10^5^ ± 8.59 · 10^4^ to 3.79 · 10^8^ ± 1.17 · 10^8^ cells L^−1^ groundwater (mean ± stdev, Fig. 1*D* and Dataset S3 *A*–*F*, 16S qPCR data corrected for gene copy number), with bacteria outnumbering archaea by one to three orders of magnitude. Consistent with the high proportion of bacteria to archaea, gene copy numbers of bacterial methane monooxygenase *pmoA* mostly exceeded those of the archaeal *mcrA* gene by one to three orders of magnitude (Fig. 1*E*), suggesting a dominant role of bacterial Type I methanotrophs in methane cycling. Gene copy numbers of *mmoX*, common in bacterial Type II methanotrophs (48), varied between wells. Neither methane concentrations nor methane oxidation rates correlated with total bacterial or archaeal abundance, but were significantly correlated with *pmoA, mcrA*, and *mmoX* gene abundances (*P* < 0.05, Spearman correlation, *SI Appendix*, Fig. S3). Methane turnover times also correlated with *pmoA*, *mcrA,* and *mmoX* gene abundances (*P* < 0.05, Spearman correlation).

**Fig. 2.**
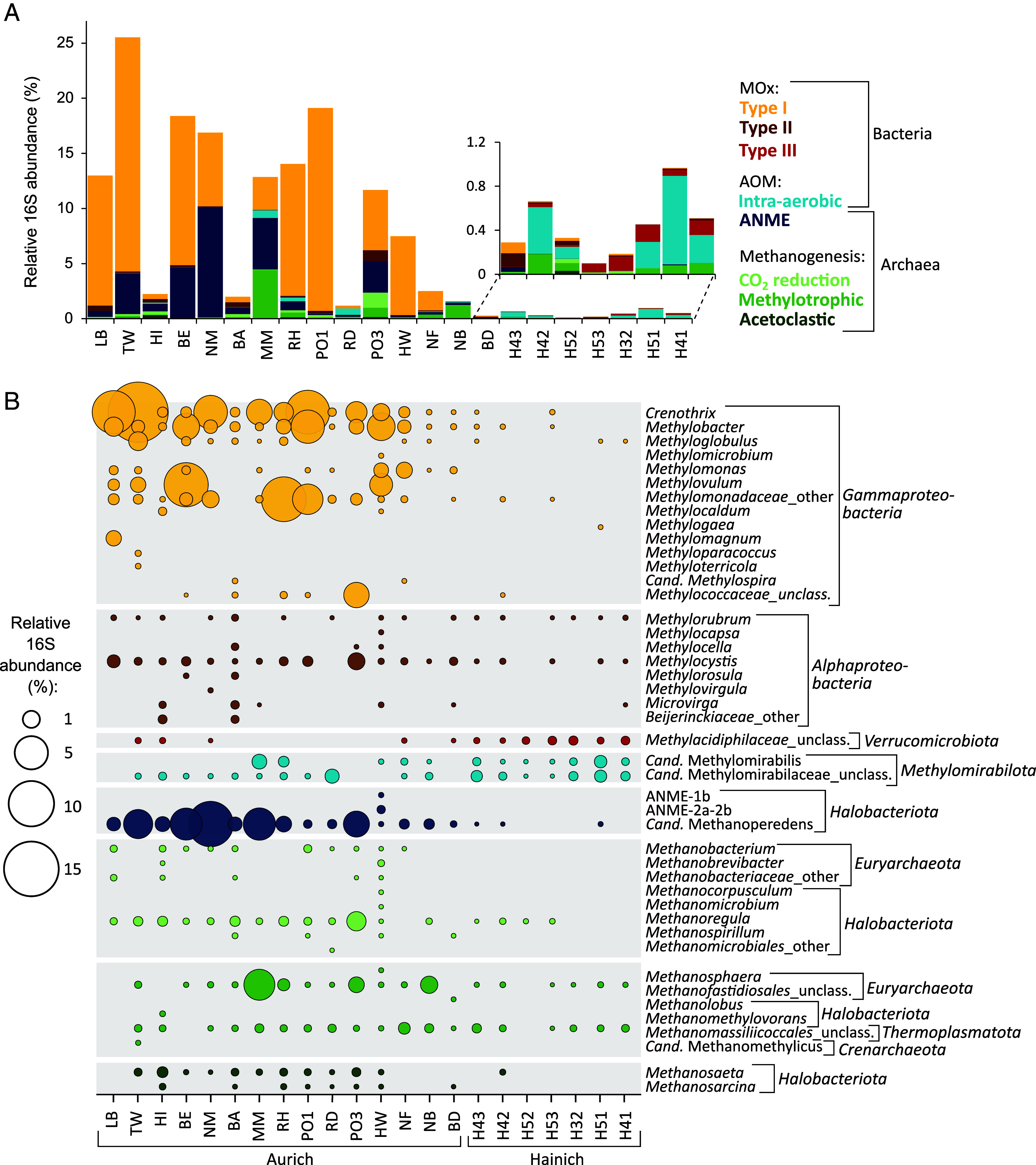
Relative abundance of microbial taxa involved in groundwater methane cycling. (*A*) Relative proportion of bacterial and archaeal methanotrophs and methanogens in Aurich and Hainich groundwater. Wells are organized from high methane oxidation rate (*Left*) to low rates (*Right*). 16S rRNA gene abundances were corrected for gene copy numbers per taxon. (*B*) Relative abundance of microbial genera involved in methane cycling. Colors refer to the functional groups of methanotrophs or methanogens. MOx: Aerobic oxidation of methane. AOM: Anaerobic oxidation of methane.

To estimate the amount of methane oxidized per microbial cell, we divided the rates by [*mcrA* + *pmoA*] gene abundances, assuming one copy per archaeal or bacterial cell, respectively. Multiple copies of the *pmoA* gene have so far only been reported in *Methylococcus species* (61), which were absent in our samples (Fig. 2). *Alphaproteobacterial* Type II methanotrophs usually have the *pmoA* gene in addition to *mmoX* (62), except for *Methylocella* or *Methyloferula species* (63). The latter was absent from our samples, but three wells contained low proportions of *Methylocella*, which we included in the per-cell rate calculation (Dataset S3*G*). Cell-specific methane oxidation rates ranged from 0.05 ± 0.01 to 44.03 ± 32.46 pgC cell^−1^ d^−1^ (0.004 to 3.7 pmol CH_4_ cell^−1^ d^−1^_,_ *SI Appendix*, Fig. S4).

### Low Rates of Methane–Carbon Assimilation.

In addition to methane oxidation to CO_2_, we quantified the assimilation of methane into microbial biomass in 9 of the 22 groundwater wells. Rates of methane–carbon fixation were lower than methane oxidation (W = 174, *P* = 0.0006, Wilcoxon rank-sum test; Fig. 1*A*), ranging from 8.61 · 10^−8^ ± 5.48 · 10^−8^ to 0.89 ± 0.64 µgC L^−1^ d^−1^ (mean ± stdev, Monte Carlo error propagation). Similarly, per-cell rates of methane assimilation were one order of magnitude lower than per-cell methane oxidation rates, ranging from 0.00002 ± 0.000009 to 0.309 ± 0.307 pgC cell^−1^ d^−1^ (0.000001 to 0.03 pmol CH_4_ cell^−1^ d^−1^_,_ *SI Appendix*, Fig. S4).

Compared to methane oxidation rates, methane–carbon assimilation rates were less strongly correlated with methane concentrations (r = 0.9833, *P* = 1.9 · 10^−6^), but more strongly correlated with *pmoA, mcrA*, and *mmoX* gene copy numbers (r = 0.7857, 0.8571, 0.9524, *P* = 0.02, 0.006, 0.0003) and bacterial abundance (r = 0.8333, *P* =0.01, Spearman correlation; *SI Appendix*, Fig. S3). With the exception of two wells, more than 99.7% of the total methane was oxidized to CO_2_ rather than fixed in the microbial biomass. Only wells MM (Aurich) and H53 (Hainich), showed a higher biomass uptake of 16.9 and 4.3%, respectively. Interestingly, methane turnover times correlated weakly with the fixation rates (r = 0.7833, *P* =0.04, Spearman correlation; *SI Appendix*, Fig. S3) as both MM and H53 had slower methane turnover times compared to the other wells analyzed for methane–carbon fixation.

### Type I Methanotrophs and ANME-2d Dominate High-Rate Groundwater Wells.

Out of a total of 23,849 microbial amplicon sequence variants (ASVs), 264 ASVs were classified as potential methanotrophs and 265 ASVs as potential methanogens (Dataset S3*H*). The relative abundances of potential methane oxidizers varied greatly between groundwater wells, ranging from 0.08 ± 0.05% in low-rate Hainich well H52 to 25.09 ± 1.18% in high-rate Aurich well TW, with the majority classified as bacterial methanotrophs (Fig. 2*A*). *Gammaproteobacterial* Type I methanotrophs reached up to 21.2 ± 1.1% of the microbial community, with *Crenothrix* and *Methylobacter species* as the most abundant representatives (Fig. 2*B*), and were positively correlated with *pmoA* gene abundances (r = 0.6333, *P* = 0.002, Spearman correlation; *SI Appendix*, Fig. S3). Archaeal methanotrophs belonged almost exclusively to *Cand.* Methanoperedens of the ANME-2d cluster, reaching relative abundances as high as 10.0 ± 0.1%. *Alphaproteobacterial* Type II and *Verrucomicrobial* Type III methanotrophs composed less than 1% of the methanotrophic communities, with *Methylocystis* and unclassified members of the *Methylacidiphilaceae* as the most prevalent genera. Potential methanotrophs of the *Methylomirabilota* were present in all but 3 groundwater wells, composing up to 0.8 ± 0.1% of the microbial community.

Groundwater methane oxidation rates and methane concentrations correlated strongly with the relative proportions of Type I methanotrophs and ANME archaea and their most abundant genera *Crenothrix*, *Methylobacter,* and *Cand.* Methanoperedens, and weakly with the relative proportions of Type II methanotrophs such as *Methylocystis* (*P* < 0.05, Spearman correlation, *SI Appendix*, Fig. S3). Unclassified Type III methanotrophs of the *Methylacidiphilaceae* and *Cand.* Methylomirabilis, although present in both the carbonate and sandy aquifers, showed a weak negative correlation with methane oxidation rates and methane concentrations, as they constituted the majority of the methane oxidizer community in the low-rate Hainich wells.

Potential methanogens were low in abundance in all samples, ranging from 0.01 ± 0.009 to 4.5 ± 0.5% of the total prokaryotic community (Fig. 2*A*). Similar to methanotroph abundance, relative proportions of methanogens were positively correlated with groundwater methane concentration (*P* < 0.01, Spearman correlation, *SI Appendix*, Fig. S3).

### High Groundwater Methane Concentrations Linked to Biogenic Origin.

**Fig. 3.**
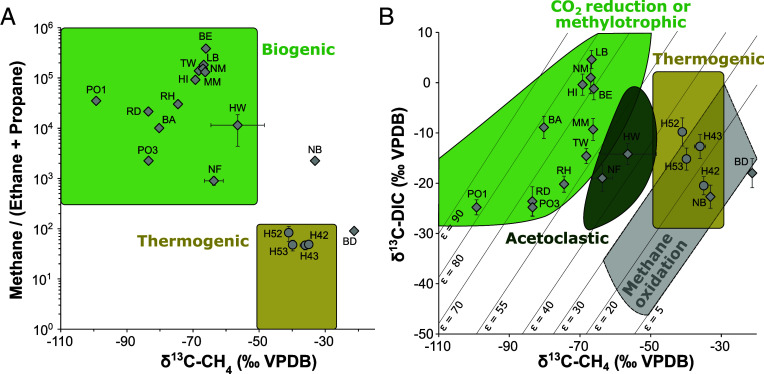
Groundwater methane isotope analysis. (*A*) Bernard diagram combining the molecular ratio of methane to [ethane + propane] and stable carbon isotopic signatures of methane. Green areas indicate typical biogenic gas and gray areas typical thermogenic gas (47). VPDB: Vienna Pee Dee Belemnite. (*B*) Differentiation of methylotrophic or CO_2_ reduction–based (green) and acetoclastic (dark green) methanogenesis from thermogenic (gray) gas based on stable carbon isotopic signatures of groundwater methane and DIC (45). DIC: Dissolved inorganic carbon. Error bars represent SD between replicate measurements combined with instrument error.

To estimate how much of the oxidized methane is net removal and how much may be recycled from methanogenesis, we analyzed methane–carbon isotopes combined with ethane and propane concentrations. Low concentrations of ethane were detected in all groundwater samples, and propane was present in seven of the wells (Fig. 1*A*). Groundwater samples with more than 60 µg L^−1^ methane showed high dryness ratios of methane to [ethane + propane] above 1,000 and light δ^13^C-signatures of less than −56.5‰ (Fig. 3 *A* and *B*), matching a biogenic gas origin (47). Young ^14^C-signatures further support a biogenic gas origin in groundwater wells with high methane concentration (*SI Appendix*, Fig. S7), as thermogenic methane would be ^14^C-free. Methane–δ^2^H signatures were consistently low (*SI Appendix*, Fig. S8), supporting a mixed biogenic origin rather than exclusive hydrogenotrophic or acetoclastic methanogenesis (45).

In contrast to the Aurich samples, dryness ratios of samples from the Hainich carbonate aquifers ranged from 11.8 ± 0.6 to 89.8 ± 0.7 (mean ± stdev), falling into the typical thermogenic window below a ratio of 100 (47). In addition, δ^13^C-signatures of methane from the Hainich site were between −34.97 ± 1.16 and −41.03 ± 1.19‰ (mean ± stdev; Fig. 3 *A* and *B*), typical of gas derived from thermal decomposition of marine organic matter (45) and within the range of measurements of natural gas from the Thuringian Basin (64). Isotope mass balance with ^14^C-DOC as biogenic and ^14^C-free as fossil end-members revealed 38.7% (well H43) to 95.5% (well H52) fossil methane in the Hainich carbonate aquifers (*SI Appendix*, Fig. S7), indicating a potential contribution of fossil methane at the low-rate sites.

### Groundwater Methane Oxidation Rates Similar to Lakes, Rivers, and Estuaries.

**Fig. 4.**
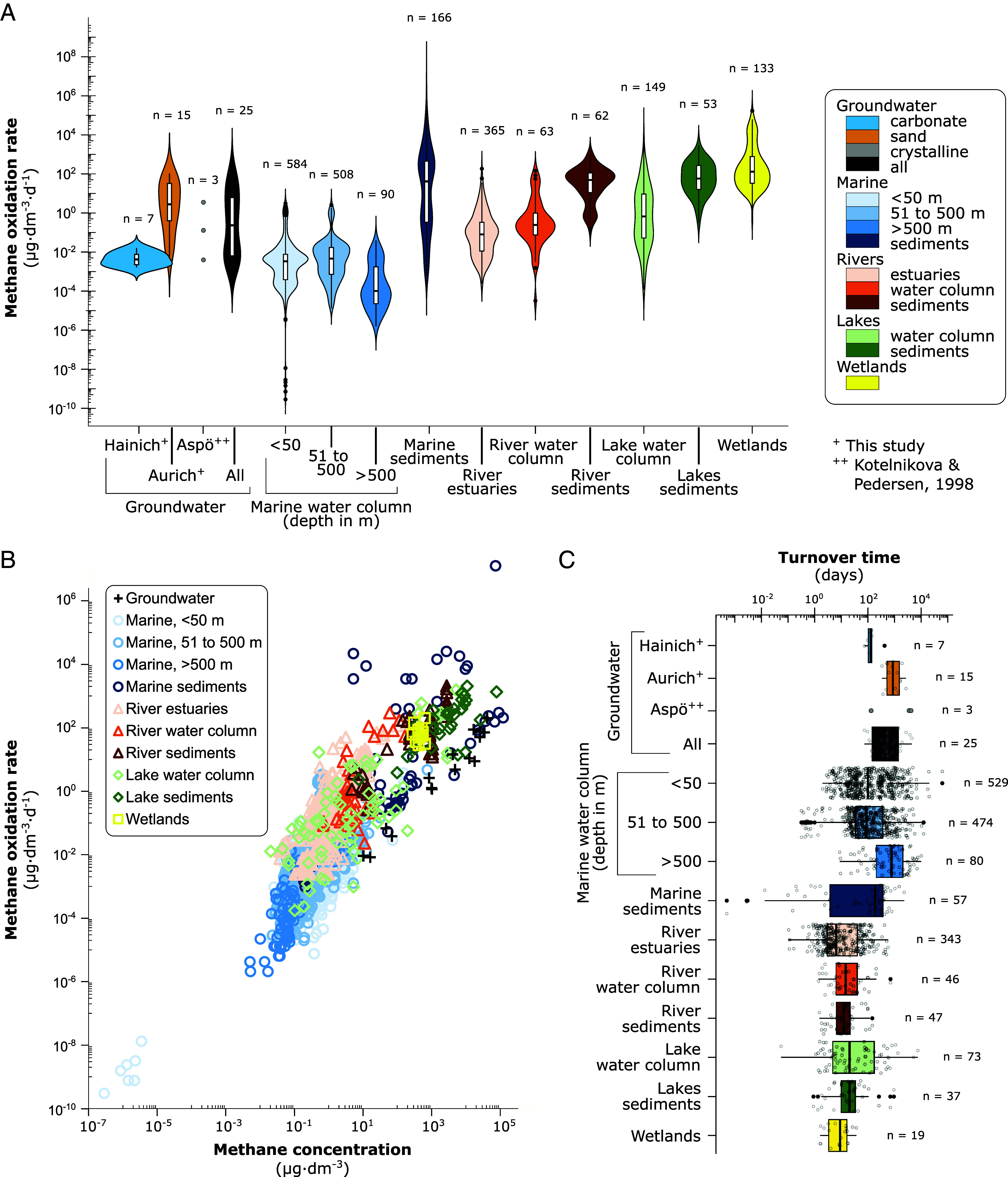
Microbial methane consumption across aquatic ecosystems. (*A*) Methane oxidation rates shown as µg methane oxidized per dm^3^ water, sediment, or, in case of wetlands, slurry, per day. Groundwater data include measurements of this study and previously published work (65). Rates and methane concentrations for other ecosystems were compiled from 70 studies including measurements across the globe (Dataset S2 *A* and *B*). (*B*) Methane oxidation rate as a function of methane concentration. (*C*) Methane turnover times calculated from methane concentrations divided by the respective oxidation rate. See *SI Appendix*, Tables S3–S7 for statistical summaries.

To understand the relevance of groundwater in the context of global methane cycling, we collected oxidation rates and methane concentrations from 70 datasets covering different aquatic ecosystems across the globe (Fig. 4 and *SI Appendix*, Table S3 and Dataset S2*A*). The only other published ^14^CH_4_ rate measurements for groundwater environments are from a deep crystalline groundwater in Sweden and ranged from 0.004 to 3.5 µg CH_4_ L^−1^ d^−1^ (65), well within the range of our measurement. The median groundwater methane oxidation rate was 0.22 µg CH_4_ L^−1^ d^−1^ (interquartile range (IQR) = 0.006, 5.81; *SI Appendix*, Table S3), falling in the same range as measurements in the water column of lakes, rivers, and estuaries (*P* = 1.0, Wilcoxon rank-sum test, Fig. 4*A* and *SI Appendix*, Table S4), and exceeding the median methane oxidation rates in the surface and deep ocean. Our highest groundwater rates even exceeded median rates of methane oxidation in lake (median = 57.74 µg CH_4_ dm^−3^ d^−1^, IQR = 16.04, 272.68; *SI Appendix*, Table S3) and river sediments (median = 48.92 µg CH_4_ · dm^−3^ · d^−1^, IQR = 11.08, 112.28), and resembled low rates from wetlands and subseafloor ecosystems.

As in groundwater, methane oxidation rates were correlated with methane concentrations in most other aquatic ecosystems (Fig. 4*B*), including the water column of lakes, rivers, and estuaries (r =0.5929, 0.7610, 0.7189, *P* = 3.5 · 10^−9^, 8.3 · 10^−10^, <2.2 · 10^−16^, Spearman correlation, *SI Appendix*, Table S5). However, wetlands, which had the highest rates of methane oxidation and methane concentration in our compiled dataset, showed no correlation between oxidation rate and methane concentration (r = −0.0737, *P* =0.7643, Spearman correlation; *SI Appendix*, Table S5). Methane turnover times in groundwater were slower than in most other aquatic ecosystems (Fig. 4*C* and *SI Appendix*, Table S6 and Dataset S2*B*) and similar to methane turnover times in deep marine waters (*P* = 1.0, Wilcoxon rank-sum test, *SI Appendix*, Table S7).

### Estimate of Global Groundwater Methane Oxidation.

**Fig. 5.**
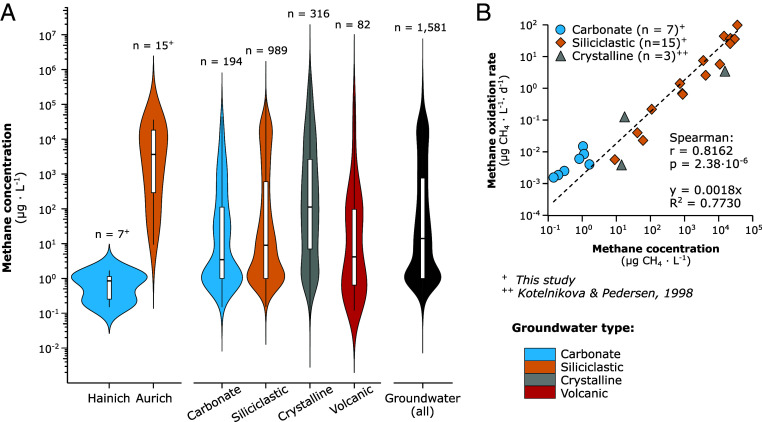
Methane concentration in global groundwater and correlation with oxidation rates. (*A*) Methane concentration in aquifers with carbonate (blue), siliciclastic (yellow), crystalline (gray), or volcanic (red) bedrock [Categories based on Gleeson et al (4)]. Concentration data were compiled from this study and measurements across the globe (Dataset S2*C*). (*B*) Correlation of groundwater methane concentration and methane oxidation rate used to estimate global groundwater methane oxidation. Covariance was tested using Spearman’s correlation.

While rate measurements are rare for groundwater environments, many studies have documented dissolved methane concentrations (5–7, 39). We combined datasets from 21 studies, including this one, from 13 countries on five continents to estimate median methane concentrations in different aquifer lithologies [Fig. 5*A* and *SI Appendix*, Fig. S9 and Table S8 and Dataset S2*C*; categories based on (4)]. The total median groundwater methane concentration was 11.27 µg L^−1^ (IQR = 1.00, 673.68 µg L^−1^, n = 1,581). Applying the strong correlation we found between methane oxidation rate and methane concentration (Fig. 5*B*), combined with a global groundwater volume of 22.6 Mio km^3^(4), we estimate 167.3 Tg CH_4_ to be oxidized by microbes year^−1^ in groundwater ecosystems (*SI Appendix*, Table S9). Separate calculation using median methane concentrations and volumes of groundwater in different bedrock lithologies resulted in 5.2 in carbonate, 41.1 in siliciclastic, 731.3 in crystalline, and 0.4 Tg CH_4_ year^−1^ in volcanic aquifers, totaling 778.0 Tg CH_4_ year^−1^. Relative to the amount of methane present, this equates to ~66% of all methane in groundwater being removed by microbial oxidation.

## Discussion

Our findings reveal that microbial oxidation in groundwater is an underappreciated methane sink, with rates comparable to those in lakes, rivers, and estuaries, and far exceeding those reported for the ocean water column (Fig. 4*A*), aligning with the broader role of microbial oxidation as an important control on upward-diffusing methane in aquatic systems (27, 29, 30). Given that the global volume of groundwater is approximately one hundred times greater than that of lakes, rivers, and wetlands combined (4), this highlights the important contribution of groundwater microbes to global methane removal. Notably, our measured rates likely represent conservative estimates, as they do not account for the abundance and activity of attached microbial communities in the subsurface, which can outnumber planktonic cells by several orders of magnitude (66). Additionally, methane oxidation may contribute to the production of small amounts of dissolved organic molecules through fermentation-based methanotrophy, a process not captured in our assay (30, 67).

The strong positive correlation between methane oxidation rates and methane concentrations (*SI Appendix*, Fig. S2) indicates that substrate availability is a key driver of microbial methane oxidation in groundwater. This relationship has also been observed in marine (68–71), lacustrine (72, 73), and riverine environments (74, 75), supporting our extrapolation of it to global groundwater. In contrast, a much weaker positive correlation between methane concentration and oxidation rate was observed in wetlands and marine sediments (Fig. 4*B*), suggesting that oxidation efficiency at high methane concentrations may be constrained by other factors, such as nutrient and electron acceptor availability, physiological limits on methane uptake rates, or differences in the substrate affinity and kinetic properties of methanotrophic bacteria and archaea.

Groundwater methane oxidation rates were weakly correlated with *pmoA, mcrA*, and *mmoX* gene abundances (*SI Appendix*, Fig. S3), matching the link between methanotroph abundance and methane oxidation activity found across aquatic ecosystems (27, 76, 77). Type I methanotrophs and members of the ANME-2d cluster were highly abundant in samples with high methane concentration and correlated strongly with methane oxidation rates (Fig. 2). *Crenothrix* and *Methylobacter species*, the most abundant Type I methanotrophs in our samples, are known for efficient methane oxidation at high methane concentrations and suboxic conditions in lakes (30, 78–83), rivers (75, 84–86), and siliciclastic aquifers (55, 56, 87). *Cand.* Methanoperedens, representing the ANME-2d cluster, is commonly found in freshwater environments with high methane, low oxygen, and dissolved iron or manganese (56, 88). In addition to high methane concentrations, groundwater from the Aurich sandy aquifers had almost no detectable oxygen and low levels of iron and manganese (*SI Appendix*, Fig. S5), likely creating optimal conditions for these organisms to dominate methane oxidation. This was also supported by the high per-cell methane oxidation rates in the Aurich samples (*SI Appendix*, Fig. S4), which far exceeded per-cell rates measured in a freshwater lake (57) and even rivaled measurements in bioreactors with *gamma*- and *alphaproteobacterial* methanotrophs under optimized conditions (89).

In contrast, uncultured methanotrophs of the *Methylacidiphilaceae* and Cand. Methylomirabilis prevailed at both high and low methane concentration, dominating the methanotroph community in the low-rate Hainich carbonate aquifers (Fig. 2). *Methylomirabilota* are known for nitrite-dependent intra-aerobic methane oxidation (51) and are common methane oxidizers in groundwater (54), lakes (30, 82, 90, 91), and wetlands (92–94). In the Hainich groundwater, nitrite is provided by ammonia oxidation or nitrate reduction (95, 96), likely fueling methane oxidation by *Cand.* Methylomirabilis. Type III methanotrophs of the *Methylacidiphilaceae* are known from acidic geothermal environments (97), but have also been detected in groundwater (90) and lake sediments (81). Both Aurich and Hainich groundwater is characterized by low temperatures and circumneutral pH (*SI Appendix*, Fig. S6), hinting at a wider environmental range of these methanotrophs that remains to be explored.

At both the Aurich and Hainich sites, methane was mostly oxidized to CO_2_, and only 0.1 to 17% was assimilated into microbial biomass, indicating that groundwater microbes use methane as an energy rather than a carbon source. Many *gammaproteobacterial* methanotrophs, including *Crenothrix sp*ecies, have been described to grow on a variety of small organic molecules in addition to methane (98), and methanotrophs of the *Methylomirabilota* and *Verrucomicrobiota* can assimilate inorganic carbon (62, 99). Nevertheless, per-cell methane–carbon fixation rates, ranging from 0.02 to 309.6 fgC cell^−1^ d^−1^, can even exceed the estimated average of 26 fgC cell^−1^ for microbes in the terrestrial subsurface (100, 101), suggesting that some groundwater methanotrophs can sustain a substantial part of their biomass carbon through methane assimilation. Studies in other aquatic ecosystems reported 22 to 64% methane–carbon assimilation, with per-cell rates of 3.2 to 482.8 fgC cell^−1^ d^−1^, in lakes (30), 44 to 56% in streams (75), and 1% to up to 80% in ocean systems (69, 102–104). Thus, the partitioning of methane-derived carbon between biomass assimilation and complete oxidation may vary with environmental factors and between aquatic methanotrophic communities.

Turnover times provide an estimate of how efficiently the groundwater microbiome acts as a filter, oxidizing methane before it is exported from groundwater to soils or to downstream aquatic ecosystems such as wetlands, lakes, or the coastal ocean. In our systems, turnover times were significantly faster at low methane concentrations in the carbonate aquifers compared to elevated methane concentrations in the sandy aquifers, ranging from 3 mo to nearly a decade (Fig. 1*C*). This is slow compared to other aquatic ecosystems (Fig. 4*C*), but whether groundwater will be a potentially important source of methane depends on water residence times, i.e., the time from groundwater recharge to discharge. The groundwater samples in this study were collected from shallow aquifers at depths ranging from 7 to 136 m below the surface (*SI Appendix*, Fig. S6). These aquifers predominantly contain modern groundwater (<50 y old) (4), with residence times spanning from few years to several decades (105, 106). If not brought to the surface through pumping, methane turnover times of less than a few years would allow the groundwater microbiome to oxidize most of the methane pool before it is exported. At low methane concentrations, such as in the Hainich aquifers, rapid methane turnover likely results in complete methane consumption, whereas aquifers with elevated methane concentrations, such as those at the Aurich site, may act as a significant methane source to soils and surface waters.

The two different geologic settings we investigated roughly spanned the range of methane concentrations found in global groundwaters (Fig. 5*A*). At both sites, the primary source of methane was biogenic, although thermogenic gas likely contributed to the dissolved methane pool in the carbonate aquifers. Historically, the additional presence of ethane or propane is an indicator of thermogenic gas (47), but recent work proposed the microbial production of trace amounts of ethane or propane alongside methanogenesis (107, 108). High DOC concentrations combined with mostly anoxic conditions likely contributed to the much higher methane concentrations found in Aurich sandy aquifers (*SI Appendix*, Fig. S5) (8). Interestingly, in most wells from this site, the ^14^C-content of the methane exceeded both that of DOC and DIC, indicating that young organic material from surface transport, rather than older organic deposits or dissolved CO_2_, was the main substrate for methanogenesis. The low abundance of potential methanogens in the groundwater itself (Fig. 2*A*) also argues that methane in the sandy aquifers may not have been produced locally, but rather transported or mixed into the groundwater from a region of higher methanogenic activity. However, the presence of overlooked or unclassified methanogens – such as recently discovered members of the *Thermoproteota* (109, 110) and *Korarchaeia* (111) – or alternative bacterial pathways for methane production, including from methylamines (108), methylphosphonates (112, 113), or detoxification of methyl radicals (114), could have contributed to in situ methane formation and may have been missed by our taxonomy-based approach.

Groundwater is a significant source of methane to surface waters (10, 13, 18, 20) and the atmosphere (5, 115). By extrapolating the strong correlation between methane concentration and microbial methane oxidation rates (Fig. 5*B*) to global groundwater methane concentrations (Fig. 5*A*), we provide a global estimate of microbial methane oxidation in groundwater (167 to 778 Tg CH_4_ y^−1^, *SI Appendix*, Table S9). Since methane turnover is more efficient at low methane concentrations and the majority of aquifers contain low methane levels (Fig. 5*A*), our estimates are likely to be conservative, underrepresenting the efficiency of the groundwater methane filter. Compared to other methane fluxes, our estimate of the groundwater methane filter represents approximately five times the global methane sink in soils and up to three times the emissions from wetlands and inland freshwaters (2). Notably, much of the uncertainty in current estimates of global methane fluxes is attributed to overestimates of emissions from these surface water systems (2). By reducing methane export to such ecosystems, the groundwater methane filter represents a sink that has not yet been adequately considered in global methane budgets.

Part of the large uncertainties in our estimates stems from the elevated median methane concentrations reported in crystalline groundwater, primarily due to high methane levels in deep granitic aquifers (116, 117). Our approach may overestimate the contribution of deep groundwater to methane transport as deep aquifers are largely disconnected from the active water cycle (118). Future studies are needed to expand the groundwater methane dataset and reduce such uncertainties. Regardless, our findings show that while a substantial fraction of methane is oxidized by the groundwater microbiome, a significant fraction remains, particularly in high-methane groundwaters, and can be transported to downstream surface waters or to the atmosphere before it can be fully oxidized. With the development of deep groundwater resources currently being discussed as a solution to the global water crisis (119), the risk of mobilizing previously isolated methane reservoirs increases. Direct exploitation of deep aquifers – through drilling and pumping – could therefore trigger the release of more methane to surface ecosystems, making it even more important to understand the groundwater methane filter.

Our study provides a global-scale estimate of microbial methane oxidation in groundwater, highlighting its role as a previously underappreciated methane sink. With recent evidence showing that global groundwater temperatures are rising due to climate change (120), and given the known temperature dependence of microbial methane production (121), groundwater methane levels may increase in the future. Our findings establish a critical baseline for future research on methane cycling in aquifers and emphasize the need to monitor methane dynamics as part of groundwater quality assessments. In particular, methane measurements in regions undergoing deep groundwater development may be essential to prevent unintended greenhouse gas emissions. Understanding the groundwater methane filter should be considered in water resource planning and policy discussions to preserve this global keystone ecosystem.

## Materials and Methods

### Site Description and Groundwater Sampling.

Groundwater was sampled from two locations: the Hainich Critical Zone Exploratory (CZE) in Central Germany (Western Thuringia) and the area around the city of Aurich in Northern Germany (Northwest Lower Saxony). Hainich groundwater flows in a fractured carbonate bedrock consisting of alternating layers of Early Triassic limestone and marlstone (122). Of the seven wells sampled, two reach the lower, oxic aquifer assemblage (H41 and H51), and five the upper, mostly anoxic aquifer assemblage (H32, H42, H43, H52, and H53). Hainich groundwater is characterized by low DOC concentrations (<1 mg L^−1^) and circumneutral pH (7.1 to 7.3) (123).

Groundwater derived from the Aurich area flows in porous aquifers embedded in glacial sand deposits and confined by layers of marine clay (124). Of the fifteen wells sampled, three reach deeper confined aquifer assemblages within Pliocene sediments (NF, PO3, and BE), and twelve reach aquifers within Pleistocene sediment deposits (BA, HI, RD, PO1, NM, TW, MM, BD, NB, RH, LB, and HW). Aurich groundwater can be rich in iron, manganese, and DOC and has a slightly acidic pH (5.1 to 7.0) (124).

Groundwater was sampled in March 2023 using submersible pumps (MP1, Grundfos, Bjerringbro, Denmark) with a flow rate of 15 ± 1 L·min^−1^. Prior to sampling, stagnant water was pumped until physicochemical parameters remained stable. All glassware used for sampling was prebaked at 500 °C for 5 h or autoclaved for 20 min at 120 °C.

### Methane Concentration and Isotope Analysis.

Groundwater samples were collected in 125 mL serum flasks, filled from bottom to top, overfilled 2x, and sealed without headspace. Following standard operating procedures for each site, Aurich samples were analyzed in duplicates, Hainich samples in triplicates. To stop microbial activity, 2 mL 37% HCl was added, and samples were stored at 4 °C. Concentrations of dissolved methane, ethane, and propane were determined using a headspace equilibrium method and analyzed on a Trace 1310 GC (Thermo Fisher Scientific, USA). Stable isotope signatures were measured for samples ≥0.0005 mg L^−1^ methane (δ^13^C) or ≥0.5 mg L^−1^ methane (δ^2^H) using an Agilent 6890 GC (for δ^13^C) or a Trace GC and Isolink/ConFlow IV (for δ^2^H) coupled to a MAT253 isotope ratio mass spectrometer (IRMS; Thermo Fisher Scientific, Waltham, MA). Details are provided in the *SI Appendix*.

Groundwater samples with ≥0.1 mg L^−1^ methane were analyzed for ^14^C-content using a headspace equilibrium method as described in the *SI Appendix*. Methane was analyzed for its ^14^C-content by accelerator mass spectrometry (AMS) at the Keck Carbon Cycle Accelerator Mass Spectrometer Facility, University of California, Irvine (125). Radiocarbon data are reported as Δ^14^C (‰) or fraction modern (FM), using the OX1 standard for decay correction to 1950. Fossil (^14^C-free) samples show a Δ^14^C of −1,000‰ or a FM of 0, while samples with a Δ^14^C ≥ 0 or a FM ≥ 1 are considered modern (^14^C-enriched).

### ^14^C-CH_4_ Incorporation Assay.

To measure methane oxidation by groundwater microbes, we used a method previously established for marine ecosystems (102) and adapted it to the low biomass in groundwater. The method takes advantage of the high sensitivity of AMS requiring only trace amounts of ^14^C-CH_4_ (0.0000003 to 0.3% of the total groundwater methane) combined with short incubation times (~24 h). Low-level labeling was used to avoid increasing in situ methane concentrations, as has been done in previous efforts (126–128). Radiocarbon tracers were prepared previously (102) and diluted with ^14^C-free CO_2_ in pre-evacuated 6 L stainless-steel canisters to final activities of 0.00023125 kBq per 50 µL of tracer (3.97 · 10^−14^ moles ^14^C in 50 µL; TRACER 1) and 0.0185 kBq per 50 µL of tracer (3.15·10^−12^ moles ^14^C in 50 µL; TRACER 2) (129). Since microbes either oxidize methane to CO_2_ (DIC pool) or incorporate it into their biomass (POC pool), two setups were used to track the ^14^C-uptake. Five bottles were collected per setup, three of which were treated with ^14^C-labeled methane (Label), and the other two served as background (Control) and killed control (Kill).

For DIC-analysis, groundwater was collected in 125 mL serum flasks, filled from bottom to top, overfilled 2x, and sealed without headspace. Except for background controls, 50 µL of TRACER 2 were added per bottle using a gas-tight Hamilton syringe equipped with a Chaney adapter (Hamilton, Reno, NV). To prevent gas escaping, the bottles were turned upside down for label addition and incubated as such at 13 ± 1 °C in the dark. Prior to tracer application, killed controls were treated with 625 µL NaN_3_ solution (50 g dissolved in 250 mL Milli-Q-water, filter-sterilized) using a 2-syringe technique and vigorously shaken for 30 s. Background controls were incubated under the same conditions as labeled samples and kills. To stop microbial activity and stabilize the DIC, 0.5 mL concentrated NaOH solution was added to each bottle after ~24 h. To remove unused ^14^C-label, bottles were opened in anoxic tents inflated with nitrogen (Erlab, Val de Reuil, France) and bubbled with nitrogen for 45 min. After bubbling, half of each sample was discarded, and bottles were resealed within the anoxic tents. For DIC headspace extraction, samples were treated with 2 mL 85% H_3_PO_4_ (final pH < 2), incubated upside down at 70 °C for 10 h, and frozen, first at −20 °C, followed by placement in a cold solution (dry ice mixed with isopropanol). The entire headspace gas was transferred to a cryogenic purification vacuum line equipped with a dry ice/isopropanol-water trap. Purified CO_2_ was trapped using liquid nitrogen and sealed into prebaked glass tubes.

For POC-analysis, groundwater was sampled and processed similar to the DIC-setup. Because of the much smaller amount of POC, 2 L borosilicate bottles were used. As biomass uptake of methane-derived carbon was expected to be small compared to the oxidation to CO_2_, POC analysis was only performed on 9 of the 22 sampled groundwater wells. Tracer addition and incubation were done similar to the DIC setup using 50 to 180 µL of TRACER 1 per label or kill bottle. Killed controls were treated with 10 mL NaN_3_ solution prior to labeling. After ~24 h, microbial activity was stopped by adding 10 mL 3 M HCl (pH < 2). To remove unused label and DIC, POC-bottles were opened and bubbled with N_2_ gas for 1 h using sterile stainless-steel needles (30 cm, autoclaved 20 min at 120 °C); followed by filtration over prebaked MN-QF-10 filters (ø 47 mm, 0.3 µm pore size, Macherey-Nagel, Düren, Germany). Filters were freeze-dried, sealed in evacuated quartz tubes with ~60 mg CuO per samples, and combusted at 900 °C for 10 h.

DIC- and POC-derived CO_2_ was purified on a cryogenic vacuum line equipped with a dry ice/ethanol- water trap and a liquid-nitrogen CO_2_-trap, followed by graphitization and ^14^C-analysis at the AMS facility in Irvine (125). To assess contamination during the DIC extraction process, samples containing 125 mL Milli-Q-water plus IAEA-C-1 and IAEA-C-2, respectively, were prepared (final concentration 90 mg · L^−1^), extracted, and analyzed for their ^14^C-content along with the DIC-samples. Blank assessment of the POC method was done in a previous study (130).

### Rate Calculation.

Rates were calculated based on the DIC and POC label incorporation (Label) minus the ^14^C-content of the DIC and POC background (Control), and the DIC concentration or amount of POC retained on the filter (mgC), respectively, relative to the groundwater volume (V_L_), incubation time (T_d_), and CH_4_ tracer activity (Eq. **1** and Dataset S1 *A*–*F*).

[1] $${\mathrm{Rate}}_{{\mathrm{mgCL}}^{-1}d^{-1}}=\frac{\left((\frac{14C}{12C}\mathrm{Label}-\frac{14C}{12C}\mathrm{Control})∙\mathrm{mgC}\right)}{\frac{14C}{12C}{\mathrm{CH}}_{4}\mathrm{tracer}∙V_{L}∙Td}.$$

As methane concentrations varied greatly between the groundwater wells, tracer activities were corrected based on the amount of methane per incubation bottle and the ^14^C-content of the groundwater methane. A Δ^14^C-CH_4_ of −500‰ was assigned to samples where ^14^C-measurement of methane was not possible. Radiocarbon values of groundwater methane from the Hainich site were derived from a previous publication (60). No correction was made for the addition of dead CO_2_ along with the ^14^C-CH_4_ tracer, which accounted for max. 0.000000000000009% of the total groundwater DIC and would have affected the results to the 11th decimal place. Error propagation was done using the Monte Carlo method (131) with random normal distribution of each parameter and 10,000 iterations, including both biological variability and analytical uncertainties. Killed controls of the POC-setup were in the same range as their unlabeled background counterparts (FM < 1), indicating no abiotic influence on the POC rate measurements. However, killed controls for the DIC-setup posed a major challenge, and a final 36.35% was subtracted for kill correction from all labeled DIC-rate samples (*SI Appendix*). Methane turnover times were calculated based on the groundwater methane concentration and the microbial methane oxidation rate (Eq. **2**). To convert methane oxidation rates in µgC L^−1^ · d^−1^ to µg CH_4_ L^−1^ · d^−1^, rates were multiplied by 1.3365, using 12.011 and 16.04236 as the molecular weights of carbon and methane, respectively (Dataset S1*G*).

[2] $$\begin{array}{c} \mathrm{Turnover}\;{\mathrm{time}}_{a}=\frac{\mu gCH_{4}L^{-1}}{\mu gCH_{4}L^{-1}d^{-1}}∙\frac{1}{365d}. \end{array}$$

### Global Estimate and Statistical Analysis.

Methane concentrations were collected from published datasets on groundwater with carbonate, siliciclastic, crystalline, and volcanic bedrock (categories based on (4); Dataset S2*C*), combined with measurements conducted in this study. Similarly, datasets on methane oxidation rates in aquatic ecosystems were collected (Dataset S2*A*) and combined with data from this study (*SI Appendix*, Table S8).

Methane oxidation rates, groundwater methane concentrations, and other parameters were correlated using the cor function (method = spearman or pearson) of the hmisc package v5.1.3 (132). Spearman correlation was used in the main analysis because the data were skewed toward low methane concentrations and oxidation rates and therefore not normally distributed. For comparison, Pearson correlation coefficients are provided in Dataset S4 *D* and *E*. Based on the correlation of methane oxidation rates with groundwater methane concentrations, median methane concentrations in groundwaters with different bedrock lithology as well as general groundwater (all combined) were converted to median methane oxidation rates, and extrapolated to the global groundwater volume published by Gleeson et al (4) (*SI Appendix*, Table S9). Since both groundwater methane concentrations and groundwater methane oxidation rates are skewed toward lower values, medians were chosen (rather than means) for global extrapolation. While this approach avoids bias from outliers, it may underestimate oxidation in systems with consistently high methane levels, introducing uncertainty at the upper end of the global estimate.

Methane oxidation rates and turnover times from different aquatic ecosystems were compared using the pairwise.wilcox.test function with *P*.adjust.method = bonferroni in R version 4.3.3 (133). Wilcoxon rank-sum tests were performed using the wilcox.test function in R. Nonparametric tests were chosen because the data were not normally distributed and displayed unequal variances across groups. Data were visualized using R packages ggplot2 v.3.5.0 (134) and scales v.1.3.0 (135).

### Groundwater Chemistry and DOC Analysis.

Groundwater DIC and DOC concentration was analyzed in triplicates on an Elementar highTOC (Elementar, Langenselbold, Germany) as described in the *SI Appendix*. Concentrations of major ions, dissolved oxygen, iron, and manganese were derived from previous publications (123, 136) and the NLWKN database.

DOC was extracted from 10 L groundwater filtered over precombusted 0.45 µm glass fiber filters as described in the *SI Appendix*. Radiocarbon measurement was carried out at UCI following established protocols (125). To estimate the contribution of methanogenesis to the groundwater methane pool, isotope mass balance was performed as detailed in the *SI Appendix*.

### DNA Extraction, 16S Sequencing, and Quantitative PCR.

Groundwater was collected in autoclaved 10 L containers (20 min, 120 °C) and filtered over 0.2 µm Supor membrane filters (PALL Corporation, Port Washington, USA) within the same day. Extraction was done in triplicate with 0.75 to 10.5 L per replicate (*SI Appendix*, Table S1) using the DNeasy PowerWater Kit (Qiagen, Hilden, Germany) with a final elution volume of 100 µL. Extraction yielded on average 5.8 ± 5.3 ng · µL^−1^ DNA. Amplicon sequencing of the microbial 16S rRNA gene was performed on a MiSeq Illumina system (Illumina Inc., San Diego, USA) using primer pair EMP_515F/EMP_926R with V4-V5 chemistry (*SI Appendix*, Table S2) (136, 137) following a two-step PCR approach as described elsewhere (138). Raw sequences were analyzed in Rv4.2.2 (133) following the DADA SOPv.1.26 (139) for quality filtering and ASV assembly as outlined previously (140). ASV taxonomy was assigned using the SILVA database v138.1 (141). ASV read numbers were corrected for taxonomic variation in rRNA operons based on rrnDB v5.8 (142) using the RDP online classifier v2.13 with a 0.8 confidence cutoff (Dataset S3*I*). Raw 16S sequence data are available at NCBI under BioProject accession PRJNA1236026.

qPCR was performed on a Mx3000P instrument (Agilent, Santa Clara, USA) using the Maxima SYBR Green Mastermix (Thermo Fisher Scientific, Germany). To estimate absolute microbial abundances, bacterial and archaeal 16S rRNA genes were quantified. To estimate the genetic potential for microbial methane oxidation, genes *mcrA,* encoding archaeal methyl coenzyme M reductase, *mmoX*, encoding the hydroxylase subunit of the soluble methane monooxygenase, and *pmoA*, encoding the small subunit of the particulate methane monooxygenase, were quantified. Details on qPCR primers, cycling conditions, and standard preparation are described in the *SI Appendix*.

## Supplementary Material

Appendix 01 (PDF)

Dataset S01 (XLSX)

Dataset S02 (XLSX)

Dataset S03 (XLSX)

Dataset S04 (XLSX)

## Data Availability

16S sequence data has been deposited in NCBI database under BioProject accession no. PRJNA1236026 (143). All other data are included in the manuscript and/or supporting information.
