# Supplementary material for: Microbial oxidation significantly reduces methane export from global groundwaters
Source: Proc Natl Acad Sci U S A. 2025 Oct 13;122(42):e2508773122. doi: 10.1073/pnas.2508773122 (PMC12557531; doi:10.1073/pnas.2508773122)
Supplement: Supplementary file 1 — Appendix 01 (PDF) [file pnas.2508773122.sapp.pdf]

Supplementary Material

Microbial oxidation significantly reduces methane export from global groundwaters

Beatrix M. Heinze[1,2,*], Valérie F. Schwab[1], Kirsten Küsel[2,3,4], Stefan Schloemer[5], Andreas Roskam[6], Xiaomei Xu[7], and Susan Trumbore[1,3,7,*]

[1]Department Biogeochemical Processes, Max-Planck-Institute for Biogeochemistry, Jena, Germany

[2]Aquatic Geomicrobiology, Institute of Biodiversity, Ecology and Evolution, Friedrich Schiller University, Jena, Germany

[3]Cluster of Excellence Balance of the Microverse, Friedrich Schiller University Jena, Jena, Germany

[4]German Center for Integrative Biodiversity Research (iDiv) Halle-Jena-Leipzig, Germany

[5]Federal Institute for Geosciences and Natural Resources (BGR), Hanover, Germany

[6]State Agency for Water Management, Coastal and Nature Conservation (NLWKN), Aurich, Germany

[7]Department of Earth System Science, University of California, Irvine, CA, USA

* Correspondence: bheinze@bgc-jena.mpg.de & trumbore@bgc-jena.mpg.de; Max Planck Institute for Biogeochemistry Jena; Hans Knöll Str. 10, 07745 Jena; Germany

Supplementary Methods

Groundwater chemistry analysis

To analyze concentrations of dissolved inorganic and organic carbon, groundwater samples were collected in triplicates in 50 mL glass vials and closed without headspace. Samples were stored at 4°C until filtration over 0.45 µm pore size and analysis on an Elementar highTOC according to the manufacturer's instructions (Elementar, Langenselbold, Germany). Concentrations of anions (SO_4^{2-}, NO_3^-), cations (Na^+, K^+, Ca^{2+}, Mg^{2+}), dissolved oxygen, iron, manganese, and water conductivity were derived from previous publications on the Hainich CZE[1,2] and the database of the State Agency for Water Management, Coastal and Nature Conservation (NLWKN) in Aurich (https://www.umweltkarten-niedersachsen.de/Umweltkarten).

Methane, ethane and propane concentration analysis

Concentrations of dissolved methane, ethane, and propane were determined using a headspace equilibrium method[3]. 25 ml of the water samples were replaced by laboratory grade Helium (5.9) and the samples equilibrated to ambient temperature. After equilibration, the total headspace pressure was measured using a pressure transducer (range 0 to 160, 0.8 kPa accuracy). The concentrations of the dissolved methane and higher hydrocarbons were calculated using the partial pressure, derived from fractional concentration (see below) and total headspace pressure, temperature of the sample, volume of headspace gas and remaining water applying the Henry's Law constant of methane. With respect to concentration measurements of dissolved methane the quantification limit for the applied SOP is ~0.5 nM. Precision based on split sample analysis for the method is ± 5%. To assess accuracy, this method has been applied to air saturated seawater (ASSW) where the concentration is in the range of 2.6 nM. Hydrocarbons in the headspace were analyzed on a Trace 1310 GC (Thermo Fisher Scientific, USA) equipped with a heated valve system and column switching. One milliliter of sample was injected into the sample loops and individual components were quantified in parallel on three channels. Methane, ethane and propane were analyzed after pre-separation (C1 through C6) from a 500 µL sample loop on a non-polar polysiloxane polymer column (Restek MX-1, 15 m, 0.28 mm ID, film thickness 3 µm), backflushing components >C7. Full separation was performed on the main 50 m Al2O3 capillary column (0.32 mm ID, film thickness 5 µm). Both columns were operated non-isothermally starting at 30°C and ending at 180°C. All components were detected on a Flame Ionization Detector (FID) with helium as carrier gas and concentrations were assessed relative to hydrocarbon standards.

Methane stable isotope analysis

The lower threshold concentration for $\delta^{13}C\text{-}CH_4$ measurements is 0.5 µg L^{-1} and for $\delta\,^{2}H\text{-}CH_4$ measurements 0.5 mg L^{-1}. The $\delta^{13}C\text{-}CH_4$ was determined using two different methods[3]. Low concentrations (< 1.6µM) require a pre-concentration and cryo-focussing of up to 8ml sample gas with liquid N_2 on a 1 m stainless steel packed column (1/16" outer diameter). Components are separated after removing the liquid N_2 and methane is combusted to CO_2 at 980 °C. Isotope ratios were measured on a MAT253 isotope ratio mass spectrometer (irMS; Thermo Fisher Scientific, Bremen, Germany). Samples with higher concentrations have been analysed on a standard GC-

irMS system, an Agilent 6890 with compound separation on a 30mPorapak column, also coupled to a MAT253. δ ^{2}H-CH_4 values were determined by a similar GC-IRMS system (TRACE GC and Isolink/-ConFlow IV coupled to a MAT 253) if methane concentrations were above 1.6μM. Methane was reduced to molecular H_2 at a temperature of 1420 °C.

Precision and accuracy are concentration dependant. For the samples from Hainich and the sample BD from Aurich, which have been measured applying a cryo-focussing of methane due to the very low concentrations, precision and accuracy of δ^{13}C-CH_4 is ±0.7‰. This is based on measurements of methane in air as daily as performance test (see also Schloemer et al., 2016[4]). All other samples have been analysed using a standard GC-irMS method as described above where the precision is 0.3‰ for δ^{13}C-CH_4 and 3‰ for δ ^{2}H-CH_4.

Methane radiocarbon isotope analysis

Groundwater samples with a methane concentration of ≥ 0.1 mg L^{-1} were additionally analyzed for ^{14}C-content. Groundwater was collected in 1 L borosilicate bottle, filled from bottom to top, sealed without headspace, and stored at 4°C. Headspaces were introduced by injecting N_2 gas via sterile tubing. Samples were shaken and allowed to settle for ~2 h. Headspace gas was transferred to evacuated 125 mL serum flasks using sterile syringes. Methane was purified on a flow-through vacuum line[5] and analyzed for its ^{14}C-content by accelerator mass spectrometry (AMS) at the Keck Carbon Cycle Accelerator Mass Spectrometer Facility, University of California, Irvine[6]. Radiocarbon data is reported as $\Delta^{14}C$ (‰) or fraction modern (FM), using the OX1 standard for decay correction to 1950. Fossil (^{14}C-free) samples show a $\Delta^{14}C$ of -1000‰ or a FM of 0, while samples with a $\Delta^{14}C \geq 0$ or a FM ≥ 1 are considered modern (^{14}C-enriched).

Killed controls of the oxidation rate measurement

Killed controls are used to assess abiotic processes influencing the ^{14}C-label uptake as well as the efficient removal of unreacted ^{14}C-CH_4. However, the chosen killing procedure can have a major influence on the outcome as some methane-oxidizing microbes have been shown to resist high temperatures[7,8], low pH[9], high pH[10–12], or harsh chemicals such as mercuric chloride and sodium azide[13,14]. Initially, we used 625 µL of a 0.2 g mL^{-1} sodium azide solution added per 125 mL groundwater sample for killing. Killed controls of the oxidation rate measurements with sodium azide as chemical killing agent had 56.5 to 937.4% higher ^{14}C-contents than the respective ^{14}C-labelled replicates without killing treatment (Figure S1). A test measurement using 625 µL of a 50% zinc chloride solution as chemical killing agent exceeded its labelled counterpart by 156.4%. High ^{14}C-contents in killed controls were also found in previous studies[15,16], ranging as high as 64% of the respective rate, indicating that an insufficient inhibition of microbial activity might be a general problem during these control measurements. In addition, killed controls exceeding the ^{14}C-content of the labelled replicates suggested chemical (i.e. abiotic) interactions influencing the ^{14}C-tracer conversion. Other causes such as tracer impurities or insufficient removal of unreacted tracer, would have affected the labeled replicates similarly and therefore cannot explain the high ^{14}C-content of the killed replicates.

To further assess the abiotic background of our DIC-based oxidation rate measurements, we prepared additional samples using either heat killing or direct killing (in this case by NaOH addition) without incubation. Both methods are commonly used in microbial metabolic rate measurements, including measurements of methane oxidation[17–21]. Additional samples (125 mL each) from groundwater well H52 were heat killed (20 min, 120°C), followed by ^{14}C-tracer addition, incubation and extraction as outlined in the methods section. Another set was treated with NaOH and bubbled with N_2 gas within 30 min after the ^{14}C-tracer addition (no 24h incubation), followed by extraction and ^{14}C-measurement as outlined before. The autoclaved sample accounted for 68.8 ± 6.9% of the ^{14}C-content of its labelled equivalents (Figure S1), indicating that the heat exposure time might not have been sufficient to fully stop microbial activity. Other studies used up to 12 h at 100°C for heat-killed controls[17] and active methane oxidation was observed in volcanic environments at temperatures up to 95°C[22], indicating that elevated heat exposure times may be necessary to stop methanotrophic activity. The zero-time blank accounted for 36.35 ± 3.6% of the labelled replicates, suggesting some abiotic background. Still, microbial activity during the brief time between label addition and further processing might also contribute to some label conversion. Furthermore, label impurities cannot be ruled out, but previous work with the same batch of ^{14}C-tracer reduces the likelihood[15,23]. We conclude the zero-time blank control to represent our best estimate of abiotic background processes. Hence, we subtracted a 36.35% background from all labeled DIC-rate samples prior to the rate calculation (Dataset S1A-E). As we assumed a similar abiotic background in all samples, this will only affect the absolute and not the relative methane oxidation rates in our study.

Groundwater DOC ^{14}C-analysis

The DOC of the Aurich wells was extracted from 10 L groundwater filtered over pre-combusted 0.45 µm glass fiber filters (500°C, 5 h). To stop microbial activity and remove inorganic carbon, concentrated HCl was added directly after sampling to a final pH ≤2 and samples were stored at 4°C. DOC was extracted by solid phase using a previously established protocol[24] optimized for groundwater samples[25]. Sample preparation for ^{14}C-analysis was done as described elsewhere[26] and ^{14}C-measurement was carried out at the AMS facility at UC Irvine using established protocols[6]. To estimate the contribution of methanogenesis to the groundwater methane pool, isotope mass balance was performed using the DOC (f_{DOC}) and fossil gas (f_{fossil}) as end members (Equation 3-5), assuming DOC to represent the substrate for *in situ* methanogenesis. Radiocarbon data was expressed as fraction modern ($F^{14}C$) with fossil methane being ^{14}C-free ($F^{14}C_{fossil}$ = 0).

$$F^{14}C_{Methane} = F^{14}C_{DOC} \cdot f_{DOC} + F^{14}C_{fossil} \cdot f_{fossil} \quad (3)$$

$$f_{DOC,\%} = \frac{F^{14}C_{Methane}}{F^{14}C_{DOC}} \cdot 100\% \quad (4)$$

$$f_{fossil,\%} = 100\% - f_{DOC,\%} \quad (5)$$

Quantitative PCR of the bacterial and archaeal 16S rRNA genes, and methane oxidation genes *pmoA*, *mcrA*, and *mmoX*

Quantitative PCR (qPCR) was performed on a Mx3000P instrument (Agilent, Santa Clara, USA) using the Maxima SYBR Green Mastermix (Thermo Fisher Scientific, Germany). For quantification of the bacterial and archaeal 16S rRNA genes, primer pairs Bac8Fmod/Bac338Rabc[27,28] and Arch21F/Arch958R[29] were used. To estimate the genetic potential for microbial methane oxidation, genes *mcrA,* encoding archaeal methyl coenzyme M reductase, *mmoX*, encoding the hydroxylase subunit of the soluble methane monooxygenase, and *pmoA*, encoding the small subunit of the particulate methane monooxygenase, were quantified using primer pairs McrA169F/McrA1360R specific for *Cand.* Methanoperedens species[30], mmoX-f/mmoX-r[31], and A189f/mb661r[32], respectively. Absolute ASV cell counts per L groundwater were calculated from relative ASV abundances (based on the corrected 16S amplicon reads), combined with absolute 16S rRNA gene abundance per sample (separately for bacteria and archaea). Error bars represent standard deviations of replicate samples per groundwater well.

Standards for quantitative PCR of the 16S RNA genes and methane oxidation genes *mcrA, mmoX*, and *pmoA* were prepared from DNA extracts of 2 to 5 different groundwater wells per gene including wells of both regions (Hainich and Aurich). Wells were chosen based on their microbial community structure to cover a diverse mixture of target organisms. Target genes were amplified by PCR for 30 cycles using 1 to 5 ng genomic DNA as template, primers specified in Table S2, and the following parameters: 15 min 95°C pre-denaturation; 95°C for 45 s, annealing for 60 s, and 72°C for 60 s; 10 min final elongation at 72°C. The following annealing temperatures were used: 55°C for 16S rRNA genes, 56°C for *pmoA* and *mmoX*, and 57°C for *mcrA*. Quality and concentration of PCR products were assessed via 1% agarose gel electrophoresis and Nanodrop 1000 measurement (Peqlab, Erlangen, Germany). PCR products were cleaned using the NucleoSpin Gel and PCR clean-up kit according to the manufacturer's instructions (Macherey-Nagel, Düren, Germany). Ligation was performed using ~100 ng of PCR product and the pGEM-T Easy Vector System (Promega, Madison, WI, USA) by overnight incubation at 4°C. Plasmids were transferred into JM109 High Efficiency Competent *Escherichia coli* Cells (Promega, Madison, WI, USA) by heat-shock transformation followed by 1.5 h incubation in sterile SOC medium at 37°C. Of each transformation reaction, 100 µL were plated on LB plates with 100 µg · mL^{-1} ampicillin, 0.5 mM IPTG, and 80 µg mL^{-1} X-Gal and incubated at 37°C overnight. Successfully transformed clones were selected by blue-white screening and quality checked by colony PCR, 1% agarose gel electrophoresis, and Sanger sequencing (Macrogen Europe, Amsterdam, Netherlands). Plasmids containing the correct target gene were extracted from respective clones using the GeneJET Plasmid Miniprep Kit (Thermo Scientific, Waltham, MA, USA). Plasmid DNA of up to 5 clones per gene was combined and diluted to a final concentration of 10^8 gene copies per µL using Milli-Q-water. To generate the calibration curve, serial 1:10 dilutions of the plasmid were prepared and analyzed along with the samples. Quantitative PCR was performed in triplicates using the cycling conditions specified above.

Supplementary Figures

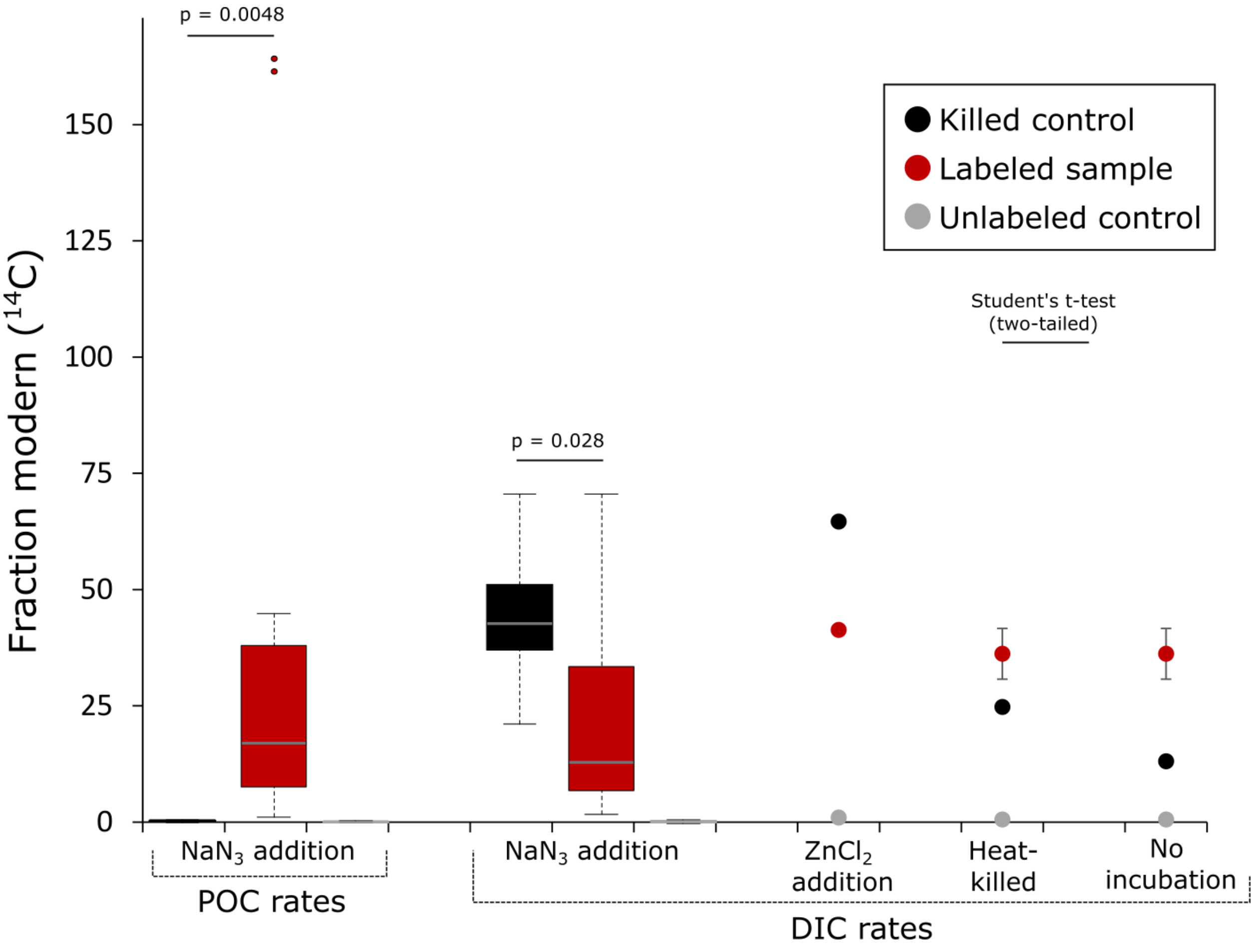

Figure S1: Radiocarbon content of killed controls (black) compared to labelled (red) or unlabeled (grey) replicates. NaN_3 samples were killed with 625 µL of 0.2 g · mL^{-1} sodium azide solution (n = 8), $ZnCl_2$ kills by addition of 625 µL 50% zinc chloride solution, heat-killing was done by autoclaving (20 min, 120°C), and the zero-time control was measured without the 24 h incubation period.

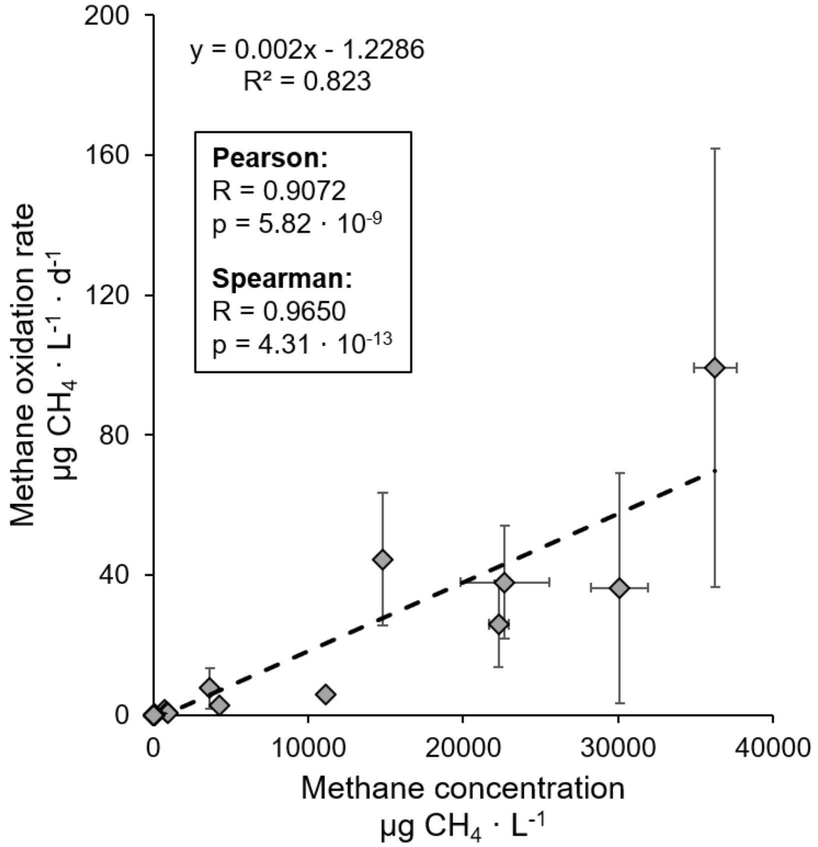

Figure S2: Correlation between groundwater methane concentration and microbial methane oxidation rate. Error bars for methane concentration represent standard deviations of two (Aurich samples, diamond shape) or three (Hainich samples, circles) replicate measurements. Error bars of methane oxidation rates represent standard deviations of Monte Carlo error propagation.

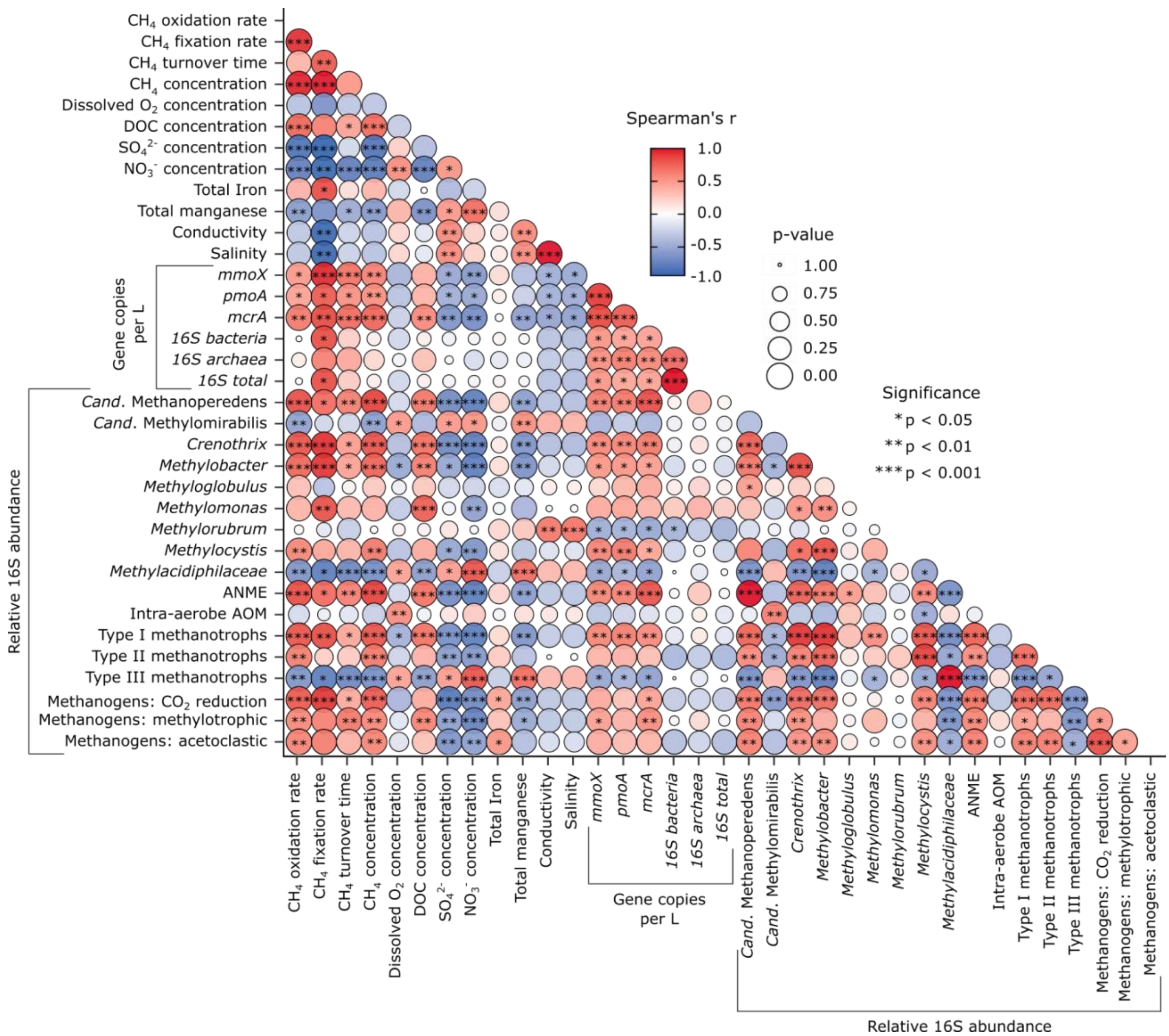

Figure S3: Spearman correlation matrix. Colors are based on Spearman's rho with red indicating positive and blue negative correlation. Bubble size refers to p value with $p < 0.05$ *, $p < 0.01$ ** and $p < 0.001$ ***. Correlation was performed for all groundwater wells combined (n = 22), except for methane carbon fixation rates, which were only analyzed in a subset of samples (n = 9).

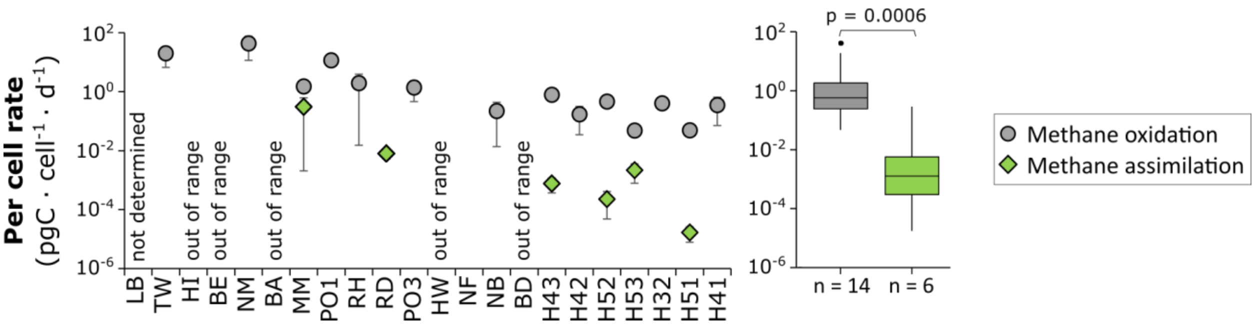

Figure S4: Cell-specific rates of methane oxidation (gray) and assimilation (green). Rates were divided by [*mcrA*+*pmoA*] gene copies per L groundwater, assuming one copy of *mcrA* or *pmoA* per archaeal or bacterial cell (Dataset S3G). Errors represent standard deviations between 3 replicate samples added to the error of the rate measurements. Similarity was tested using the Wilcoxon rank sum test.

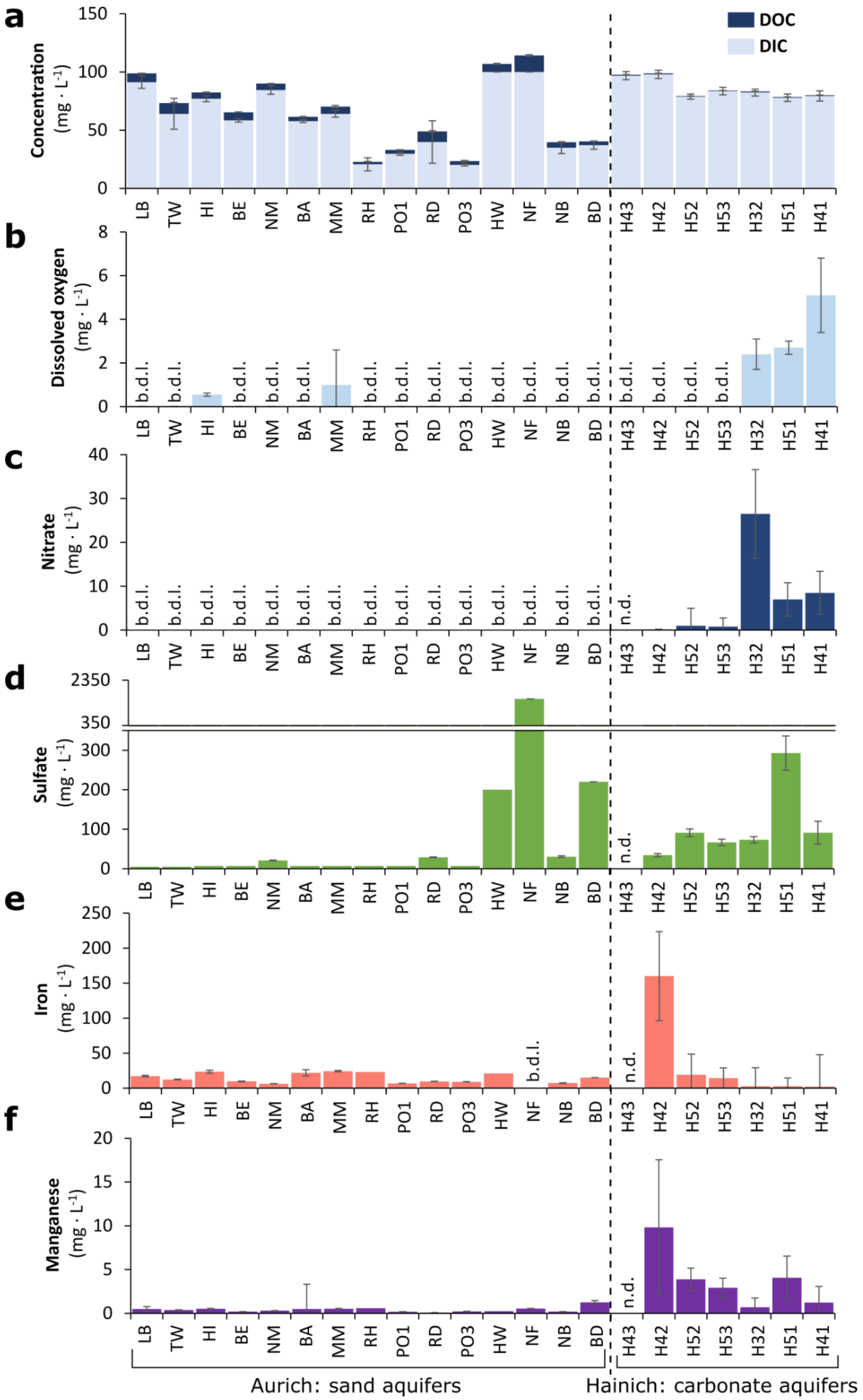
a
b
c
d
e
f
DOC
DIC
Concentration (mg · L-1)
Dissolved oxygen (mg · L-1)
Nitrate (mg · L-1)
Sulfate (mg · L-1)
Iron (mg · L-1)
Manganese (mg · L-1)
LB TW HI BE NM BA MM RH PO1 RD PO3 HW NF NB BD H43 H42 H52 H53 H32 H51 H41
b.d.l.
n.d.
Aurich: sand aquifers
Hainich: carbonate aquifers

Figure S5: Hydrochemical parameters of groundwater from carbonate and sandy aquifers in central (Hainich) and northern (Aurich) Germany. Concentrations of Dissolved inorganic carbon (DIC) and Dissolved organic carbon (DOC, a) were measured at the Max Planck Institute for Biogeochemistry in Jena. Error bars represent standard deviations of triplicate groundwater samples. Concentrations of dissolved oxygen (b), nitrate (c), sulfate (d), iron (e) and manganese (f) were collected from previous publication[1] and the database of the State Agency for Water Management, Coastal and Nature Conservation (NLWKN) in Aurich (https://www.umweltkarten-niedersachsen.de/Umweltkarten). Error bars represent standard deviations of measurements from the past five years. Not determined: n.d.; Below detection limit: b.d.l.

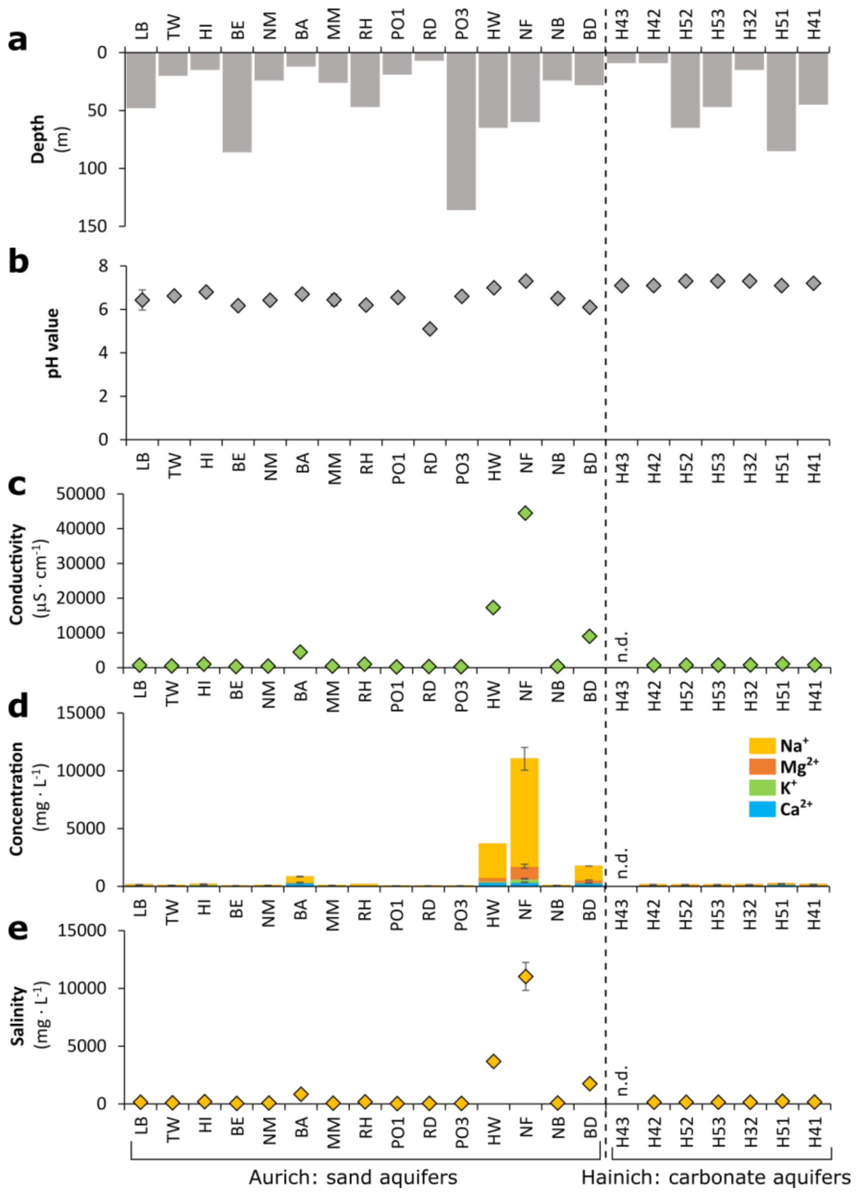

Figure S6: Hydrochemical parameters of groundwater from carbonate and sandy aquifers in central (Hainich) and northern (Aurich) Germany. Data on well depth (a), pH values (b), conductivity (c) and ion concentrations (d) were collected from previous publication[1,2] and the database of the State Agency for Water Management, Coastal and Nature Conservation (NLWKN) in Aurich (https://www.umweltkarten-niedersachsen.de/Umweltkarten). Error bars represent standard deviations of measurements from the past five years. Salinity (e) was calculated from the sum of sodium, magnesium, potassium, and calcium concentrations. Not determined: n.d.; Below detection limit: b.d.l.

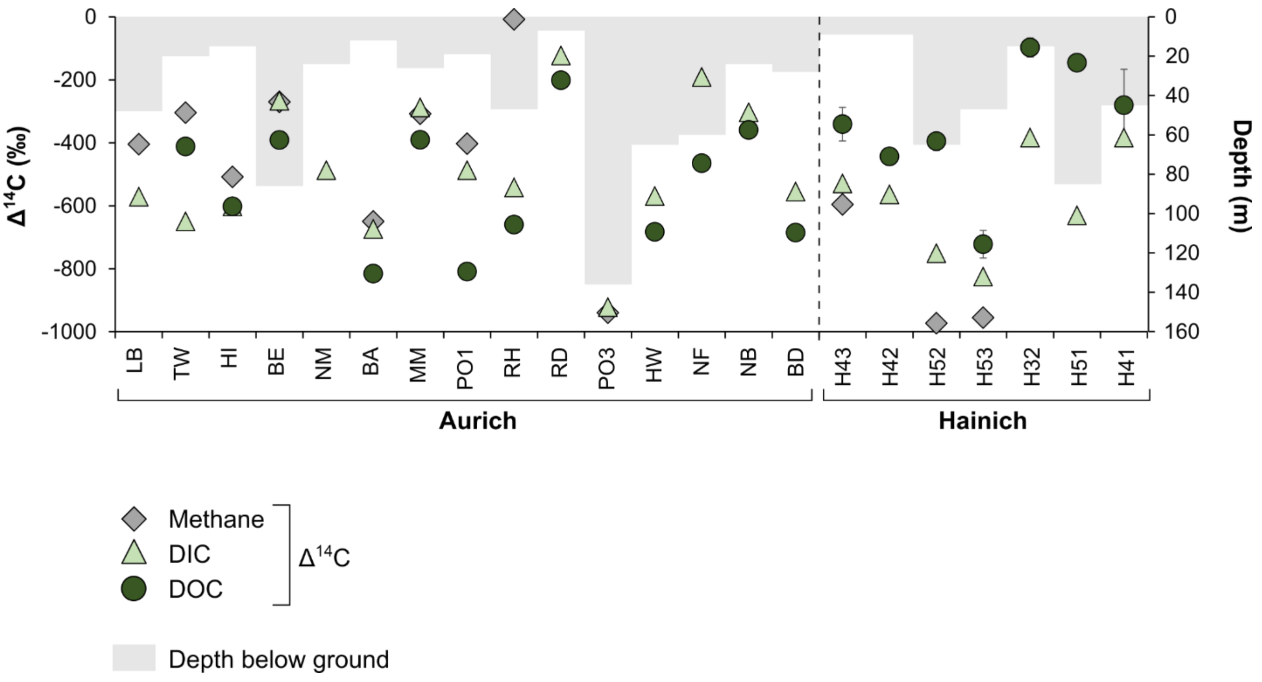

Figure S7: Groundwater well depth and radiocarbon isotopic signatures of groundwater methane, DIC and DOC. DIC: Dissolved inorganic carbon, DOC: Dissolved organic carbon. Error bars represent standard deviations between replicate measurements combined with instrument error.

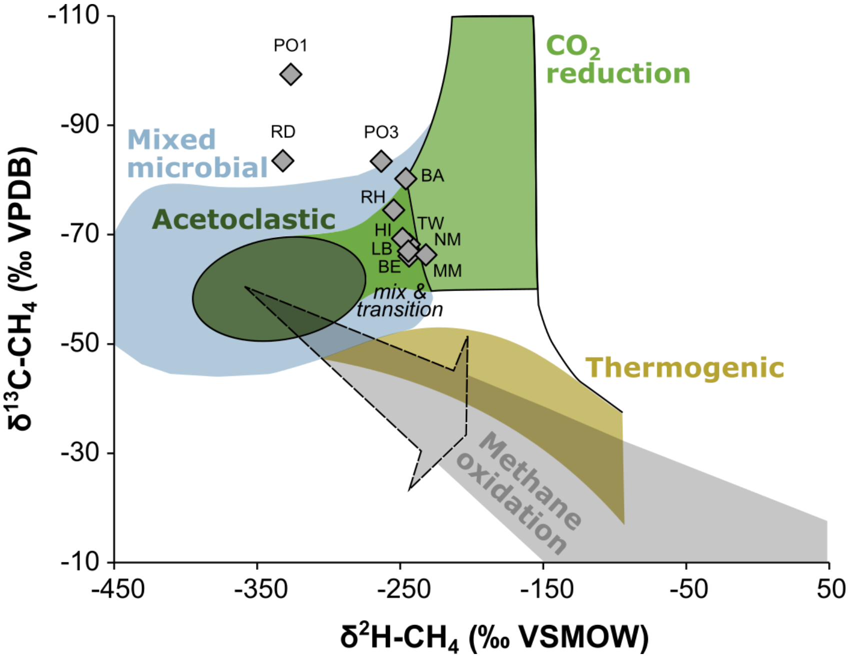

Figure S8: Differentiation of methane origin based on stable carbon and deuterium methane isotopic signatures. VSMOW: Vienna Standard Mean Ocean Water. Deuterium signatures were only analyzed in groundwater samples with high methane concentration. Error bars represent standard deviations between replicate measurements combined with instrument error.

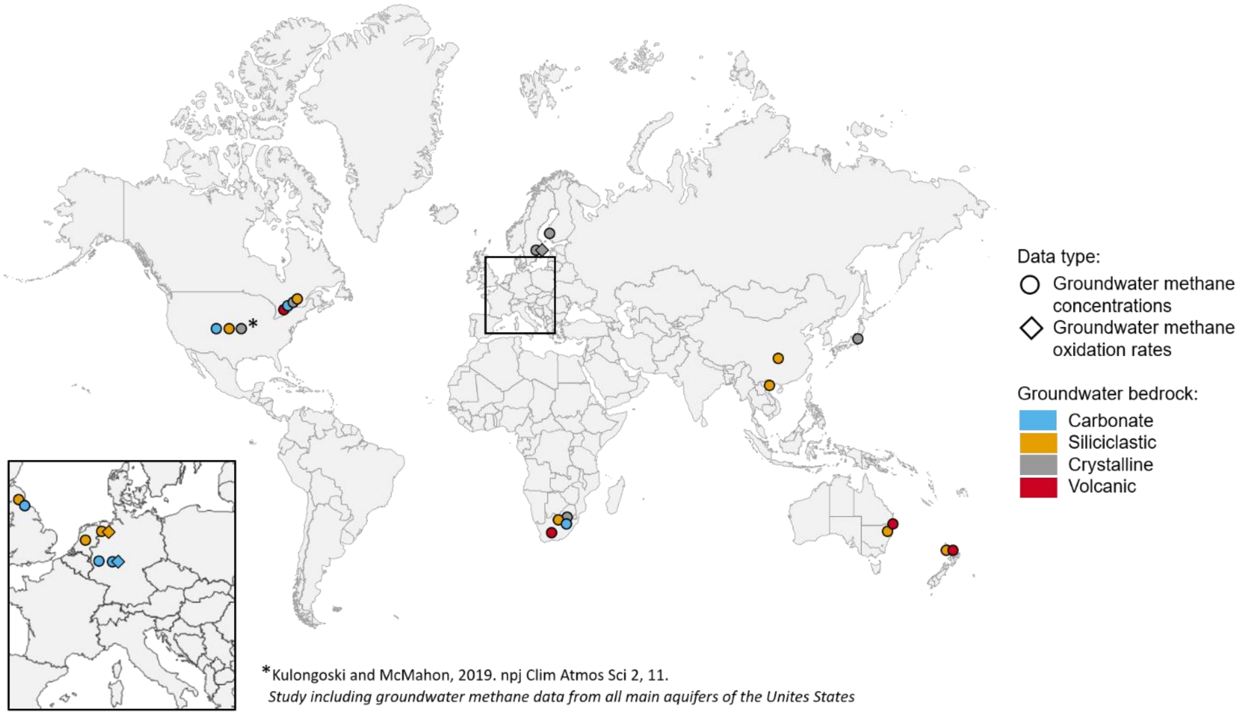

Figure S9: World map depicting the origin of groundwater methane concentrations (circle) and methane oxidation rates (diamond) used for global extrapolation. Colors refer to the aquifer lithology according to Gleeson et al.[33]. See Supplementary Dataset S2C for the data collection.

Supplementary Tables

Table S1: Groundwater volume used for DNA extraction and DNA concentration in the 100 µL eluate. DNA concentration was measured using the Qubit dsDNA Assay-Kit (Thermo Fisher Scientific, Waltham, MA, USA). Groundwater was filtered over 0.2 µm pore size.

Groundwater well	Replicate	Filtered groundwater volume (L)	DNA concentration (ng · µL^{-1})
**PO1**	a	2.0	0.6
	b	2.0	0.7
	c	1.0	0.8
**PO3**	a	3.5	0.9
	b	2.5	0.5
	c	2.5	0.7
**NM**	a	5.0	4.1
	b	4.0	5.4
	c	3.0	4.5
**NF**	a	5.0	3.7
	b	3.0	2.8
	c	2.0	1.9
**BD**	a	6.5	4.7
	b	5.0	10.1
	c	5.0	12.3
**HW**	a	4.0	n.d.
	b	4.0	n.d.
	c	4.0	n.d.
**RH**	a	4.0	1.29
	b	4.0	n.d.
	c	4.0	1.14
**HI**	a	2.0	4.9
	b	2.0	0.09
	c	2.0	0.08
**TW**	a	1.5	1.7
	b	1.5	1.6
	c	1.5	1.2
**BA**	a	4.0	19.6
	b	4.0	12.7
	c	4.0	9.2
**BE**	a	2.0	7.2
	b	1.0	5.2
	c	1.0	6.7
**MM**	a	1.5	8.7
	b	0.75	3.7
	c	0.75	3.1
**NB**	a	1.5	2.3
	b	1.0	1.3
	c	1.0	1.3
**RD**	a	4.0	14.3
	b	4.0	11.5

	c	4.0	12.1
**H32**	a	7.0	4.2
	b	6.2	4.7
	c	5.9	2.9
**H41**	a	6.5	3.1
	b	8.0	3.6
	c	8.5	5.0
**H42**	a	7.4	2.1
	b	7.6	2.3
	c	10.5	2.0
**H43**	a	8.1	9.0
	b	5.5	8.1
	c	5.7	6.7
**H51**	a	8.5	3.7
	b	6.2	n.d.
	c	5.7	1.2
**H52**	a	7.0	10.3
	b	6.6	17.0
	c	7.0	16.7
**H53**	a	6.5	18.7
	b	6.2	15.3
	c	7.0	15.7

Table S2: Primer sequences used for DNA amplification. Primers of the earth microbiome project (EMP) were used for 16S amplicon sequencing. The remaining primers were used for quantitative PCR.

Primer pair	Target gene	Sequence	Organisms	Reference
EMP_515F	16S rRNA gene	5'-gtgycagcmgccgcggtaa-3'	Bacteria and archaea	Parada et al., 2016
EMP_926R		5'-ccgycaattymtttragttt-3'		Quince et al., 2011
Bac8Fmod	16S rRNA gene	5'-agagtttgatymtggctcag-3'	General bacteria	Loy et al., 2002
Bac338Rabc		5'-gcwgccwcccgtaggwgt-3'		Daims et al., 1999
Arch21F	16S rRNA gene	5'-ttccggttgatccygccgga-3'	General archaea	DeLong, 1992
Arch958R		5'-yccggcgttgamtccaatt-3'		
McrA169F	*mcrA*	5'-gcagcaatcaccaagaagagagg-3'	Cand. *Methanoperedens*	Vaksmaa et al., 2017
McrA1360R		5'-tgcctctttgtggaggtacatgga-3'		
A189f	*pmoA*	5'-ggngactgggacttctgg-3'	Type I and II methanotrophs	Costello & Lidstrom, 1999
mb661r		3'-ccattyctgcaacgmggcc-5'		
mmoX-f	*mmoX*	5'-tcaacaccgatctsaacaacg-3'	*Methylosinus*, general	Knapp et al., 2007
mmoX-r		5'-tccagattccrccccaatcc-3'		

Table S3: Methane oxidation rates (µg CH_4 · dm^{-3} · d^{-1}) across aquatic ecosystems. Rates are shown as µg methane oxidized per dm^3 water, sediment or, in case of wetlands, slurry, per day. Groundwater methane oxidation rates include measurements of this study (Hainich, carbonate and Aurich, sand), and previously published rates[34]. Rates for other ecosystems were compiled from 72 studies including measurements across the globe (Dataset S2A).

Ecosystem type	n	Q1	Q2 (MEDIAN)	Q3	Source
Groundwater					
Hainich, carbonate	7	0.002	0.004	0.007	*This study*
Aurich, sand	15	0.44	2.61	31.14	*This study*
Aspö, crystalline	3	0.07	0.13	1.83	[34]
All	25	0.006	0.22	5.81	*This study*; [34]
Marine water column					
0 to 50 m depth	584	0.0003	0.003	0.007	[9,17,21–32]
51 to 500 m depth	508	0.0005	0.004	0.02	[15,21,38,39,42,43,46–48]
> 500 m depth	90	0.00002	0.0001	0.002	[15,21,46,48]
Marine sediments	166	0.28	27.89	417.04	[49–60]
River estuaries	365	0.01	0.08	0.32	[35,36,61–63]
River water column	63	0.08	0.24	1.00	[63–67]
River sediments	62	11.08	48.92	112.28	[65,68–70]
Lake water column	149	0.02	0.32	8.02	[35,63,67,71–78]
Lake sediments	53	16.04	57.74	272.68	[79–83]
Wetlands	133	33.13	130.73	776.02	[84–99]

Table S4: Pairwise comparison of methane oxidation rates across global aquatic ecosystems using Wilcoxon rank sum test. P values were adjusted using the Bonferroni correction. Significant comparisons ($p > 0.05$) have a gray background. $p > 0.05$ n.s.; $p < 0.05$ *, $p < 0.01$ **, $p < 0.001$ ***

Wilcoxon rank-sum test		Ground-Water n = 25	Marine water column < 50m depth n = 584	51-500m depth n = 508	>500m depth n = 90	Marine sediments n = 166	River estuaries n = 365	River water column n = 63	River sediments n = 62	Lake water column n = 149	Lake sediments n = 53
Marine water column	<50 m depth	$p = 5.24 \cdot 10^{-7}$ ***	-	-	-	-	-	-	-	-	-
	51-500 m depth	$p = 1.07 \cdot 10^{-5}$ ***	$p = 0.31$ n.s.	-	-	-	-	-	-	-	-
	>500 m depth	$p = 1.77 \cdot 10^{-9}$ ***	$p = 2.4 \cdot 10^{-10}$ ***	$p = 8.24 \cdot 10^{-13}$ ***	-	-	-	-	-	-	-
Marine sediments		$p = 0.024$ *	$p = 3.11 \cdot 10^{-60}$ ***	$p = 2.5 \cdot 10^{-56}$ ***	$p = 6.26 \cdot 10^{-33}$ ***	-	-	-	-	-	-
River estuaries		$p = 1$ n.s.	$p = 7.41 \cdot 10^{-64}$ ***	$p = 1.13 \cdot 10^{-51}$ ***	$p = 1.7 \cdot 10^{-34}$ ***	$p = 1.07 \cdot 10^{-26}$ ***	-	-	-	-	-
River water column		$p = 1$ n.s.	$p = 2.9 \cdot 10^{-24}$ ***	$p = 2.73 \cdot 10^{-23}$ ***	$p = 2.51 \cdot 10^{-21}$ ***	$p = 6.54 \cdot 10^{-6}$ ***	$p = 0.005$ **	-	-	-	-
River sediments		$p = 1.14 \cdot 10^{-5}$ ***	$p = 1.41 \cdot 10^{-35}$ ***	$p = 4.5 \cdot 10^{-35}$ ***	$p = 1.22 \cdot 10^{-23}$ ***	$p = 1$ n.s.	$p = 4.70 \cdot 10^{-29}$ ***	$p = 3.19 \cdot 10^{-13}$ ***	-	-	-
Lake water column		$p = 1$ n.s.	$p = 1.56 \cdot 10^{-31}$ ***	$p = 1.44 \cdot 10^{-28}$ ***	$p = 4.42 \cdot 10^{-20}$ ***	$p = 1.98 \cdot 10^{-8}$ ***	$p = 0.001$ **	$p = 1$ n.s.	$p = 1.33 \cdot 10^{-12}$ ***	-	-
Lake sediments		$p = 2.07 \cdot 10^{-6}$ ***	$p = 2.39 \cdot 10^{-31}$ ***	$p = 5.57 \cdot 10^{-31}$ ***	$p = 1.93 \cdot 10^{-21}$ ***	$p = 1$ n.s.	$p = 1.69 \cdot 10^{-27}$ ***	$p = 9.19 \cdot 10^{-14}$ ***	$p = 1$ n.s.	$p = 5.22 \cdot 10^{-14}$ ***	-
Wetlands n = 133		$p = 4.18 \cdot 10^{-10}$ ***	$p = 1.66 \cdot 10^{-70}$ ***	$p = 1.4 \cdot 10^{-68}$ ***	$p = 8.9 \cdot 10^{-35}$ ***	$p = 0.0005$ ***	$p = 2.15 \cdot 10^{-61}$ ***	$p = 2.02 \cdot 10^{-23}$ ***	$p = 0.002$ **	$p = 5.17 \cdot 10^{-32}$ ***	$p = 0.6$ n.s.

Table S5: Correlation analysis of methane oxidation rates and methane concentrations across aquatic ecosystems. Groundwater methane oxidation rates and concentrations include measurements of this study (Hainich, carbonate and Aurich, sand), and previously published data[34]. Data for other ecosystems was compiled from 72 studies including measurements across the globe (Dataset S2A).

Ecosystem type	n	R^2	Pearson's R	Pearson's p-value	Spearman's R	Spearman's p-value
Groundwater	25	0.7730	0.645012	$4.993 \cdot 10^{-4}$	0.8161538	$2.377 \cdot 10^{-6}$
Marine water column						
0 to 50 m depth	529	0.1118	0.33431	$2.806 \cdot 10^{-15}$	0.437595	$<2.2 \cdot 10^{-16}$
51 to 500 m depth	475	0.1009	0.31767	$1.345 \cdot 10^{-12}$	0.5857003	$<2.2 \cdot 10^{-16}$
> 500 m depth	80	0.4382	0.66193	$2.301 \cdot 10^{-11}$	0.7172877	$7.164 \cdot 10^{-14}$
Marine sediments	57	0.1261	0.3551	0.007	0.641011	$5.947 \cdot 10^{-8}$
River estuaries	343	0.9159	0.95702	$2.25 \cdot 10^{-185}$	0.7188972	$<2.2 \cdot 10^{-16}$
River water column	46	0.9218	0.96008	$5.623 \cdot 10^{-26}$	0.7609983	$8.342 \cdot 10^{-10}$
River sediments	47	0.4996	0.70685	$2.81 \cdot 10^{-8}$	0.7874402	$5.185 \cdot 10^{-11}$
Lake water column	73	0.7115	0.84353	$7.57 \cdot 10^{-21}$	0.5928681	$3.522 \cdot 10^{-9}$
Lake sediments	37	0.2589	0.50883	0.001	0.7241318	$4.066 \cdot 10^{-7}$
Wetlands	19	0.0124	0.1113	0.65	-0.0736842	0.7643

Table S6: Methane turnover times (days) across aquatic ecosystems. Turnover times are displayed in days and were calculated from methane oxidation rates and methane concentrations (Dataset S2B). Groundwater methane oxidation rates and concentrations include measurements of this study (Hainich, carbonate and Aurich, sand) and previously published rates[34]. Rates and concentrations for other ecosystems were compiled from 70 studies including measurements across the globe.

Ecosystem type	n	Q1	Q2 (MEDIAN)	Q3	MEAN	STDEV
Groundwater						
Hainich, carbonate	7	103	124	138	156	117
Aurich, sand	15	514	885	1,507	1,070	678
Aspö, crystalline	3	1,948	3,755	4,104	2,783	2,315
All	25	141	524	1,390	1,020	1,152
Marine water column						
0 to 50 m depth	529	26	100	562	729	3,171
51 to 500 m depth	474	34	88	356	413	1,022
> 500 m depth	80	208	767	2,056	1,514	2,002
Marine sediments	57	4	186	370	279	396
River estuaries	343	3	6	40	36	76
River water column	46	6	15	40	47	109
River sediments	47	7	13	22	24	33
Lake water column	73	5	21	175	319	1,068
Lake sediments	37	10	21	35	73	189
Wetlands	19	3	9	16	11	9

Table S7: Pairwise comparison of methane turnover times across global aquatic ecosystems using Wilcoxon rank sum test. P values were adjusted using the Bonferroni correction. Significant comparisons ($p > 0.05$) have a gray background. $p > 0.05$ n.s.; $p < 0.05$ *, $p < 0.01$ **, $p < 0.001$ ***

Wilcoxon rank-sum test		Ground-Water n = 25	Marine water column			Marine sediments n = 57	River estuaries n = 343	River water column n = 46	River sediments n = 47	Lake water column n = 73	Lake sediments n = 37
			< 50m depth n = 529	51-500m depth n = 474	>500m depth n = 80						
Marine water column	<50 m depth	p = 0.003 **	-	-	-	-	-	-	-	-	-
Marine water column	51-500 m depth	p = 0.0001 ***	p = 1 n.s.	-	-	-	-	-	-	-	-
Marine water column	>500 m depth	p = 1 n.s.	p = $3.67 \cdot 10^{-9}$ ***	p = $1.43 \cdot 10^{-11}$ ***	-	-	-	-	-	-	-
Marine sediments		p = 0.007 **	p = 1 n.s.	p = 1 n.s.	p = $7.47 \cdot 10^{-6}$ ***	-	-	-	-	-	-
River estuaries		p = $1.79 \cdot 10^{-13}$ ***	p = $1.26 \cdot 10^{-58}$ ***	p = $2.43 \cdot 10^{-55}$ ***	p = $7.3 \cdot 10^{-34}$ ***	p = 0.0001 ***	-	-	-	-	-
River water column		p = $8.19 \cdot 10^{-9}$ ***	p = $1.02 \cdot 10^{-8}$ ***	p = $1.66 \cdot 10^{-9}$ ***	p = $3.44 \cdot 10^{-14}$ ***	p = 0.18 n.s.	p = 1 n.s.	-	-	-	-
River sediments		p = $1.31 \cdot 10^{-9}$ ***	p = $1.04 \cdot 10^{-12}$ ***	p = $4.81 \cdot 10^{-14}$ ***	p = $1.26 \cdot 10^{-15}$ ***	p = 0.04 *	p = 1 n.s.	p = 1 n.s.	-	-	-
Lake water column		p = $1.02 \cdot 10^{-5}$ ***	p = 0.0002 ***	p = 0.001 **	p = $3.67 \cdot 10^{-11}$ ***	p = 1 n.s.	p = 0.006 **	p = 1 n.s.	p = 1 n.s.	-	-
Lake sediments		p = $2.64 \cdot 10^{-10}$ ***	p = $5.29 \cdot 10^{-6}$ ***	p = $4.11 \cdot 10^{-7}$ ***	p = $4.59 \cdot 10^{-11}$ ***	p = 0.71 n.s.	p = 1 n.s.	p = 1 n.s.	p = 1 n.s.	p = 1 n.s.	-
Wetlands n = 19		p = $1.29 \cdot 10^{-10}$ ***	p = $8.21 \cdot 10^{-8}$ ***	p = $3.12 \cdot 10^{-8}$ ***	p = $9.28 \cdot 10^{-9}$ ***	p = 0.29 n.s.	p = 1 n.s.	p = 1 n.s.	p = 1 n.s.	p = 1 n.s.	p = 0.66 n.s.

Table S8: Methane concentration (mg·L^{-1}) in global groundwater. Methane concentrations were compiled from 20 datasets including groundwater with different bedrock lithologies and locations across the globe (Dataset S2C). Hainich and Aurich refers to methane concentrations measured in this study.

Groundwater type	n	Q1	Q2 (MEDIAN)	Q3	MEAN	STDEV	Source
Aurich, sand	15	0.3	3.65	16.76	9.96	12.45	*This study*
Hainich, carbonate	7	0.0003	0.0009	0.001	0.0008	0.0006	*This study*
Carbonate	194	0.001	0.004	0.11	1.22	5.04	*This study,* [100–104]
Siliciclastic, sand	794	0.001	0.008	0.77	3.69	8.96	*This study,* [4,101,105–108]
Siliciclastic, rock	161	0.005	0.04	0.43	3.79	13.71	[100–103,109,110]
Siliciclastic, all	955	0.001	0.008	0.72	3.71	9.88	-
Crystalline	306	0.004	0.09	2.41	17.32	70.03	[34,101,102,111–116]
Volcanic	73	0.000	0.0007	0.001	3.06	19.51	[102,106,109,117,118]
Groundwater, all	1,537	0.001	0.01	0.67	6.09	33.07	-

Table S9: Estimation of methane consumed through microbial methane oxidation in global groundwater. Median methane concentrations were calculated based on compiled datasets (Tab. S8). Global groundwater volumes were taken from Gleeson et al[33].

Groundwater type	CH_4 conc.	Groundwater volume	Total CH_4	CH_4 oxidation rate		CH_4 oxidized
	µg · L^{-1}	L	Tg	µg CH_4 · L^{-1} · d^{-1}	µg CH_4 · L^{-1} · a^{-1}	Tg
Carbonate	3.50	$2.26 \cdot 10^{18}$	7.91	0.006	2.30	5.20
Siliciclastic	9.23	$6.78 \cdot 10^{18}$	62.61	0.02	6.07	41.13
Crystalline	87.92	$1.27 \cdot 10^{19}$	1,113.01	0.16	57.76	731.25
Volcanic	0.70	$9.00 \cdot 10^{17}$	0.63	0.001	0.46	0.41
SUM	-	-	1,184.16	-	-	777.99
All	11.27	$2.26 \cdot 10^{19}$	254.69	0.02	7.40	167.33

Supplementary references

1. Lehmann, R. & Totsche, K. U. Multi-directional flow dynamics shape groundwater quality in sloping bedrock strata. *J. Hydrol.* **580**, 124291 (2020).
2. Kohlhepp, B. *et al.* Aquifer configuration and geostructural links control the groundwater quality in thin-bedded carbonate–siliciclastic alternations of the Hainich CZE, central Germany. *Hydrol. Earth Syst. Sci.* **21**, 6091–6116 (2017).
3. Schloemer, S., Oest, J., Illing, C. J., Elbracht, J. & Blumenberg, M. Spatial distribution and temporal variation of methane, ethane and propane background levels in shallow aquifers – A case study from Lower Saxony (Germany). *J. Hydrol. Reg. Stud.* **19**, 57–79 (2018).
4. Schloemer, S., Elbracht, J., Blumenberg, M. & Illing, C. J. Distribution and origin of dissolved methane, ethane and propane in shallow groundwater of Lower Saxony, Germany. *Appl. Geochem.* **67**, 118–132 (2016).
5. Pack, M. A., Xu, X., Lupascu, M., Kessler, J. D. & Czimczik, C. I. A rapid method for preparing low volume CH 4 and CO 2 gas samples for 14 C AMS analysis. *Org. Geochem.* **78**, 89–98 (2015).
6. Xu, X. *et al.* Modifying a sealed tube zinc reduction method for preparation of AMS graphite targets: Reducing background and attaining high precision. *Nucl. Instrum. Methods Phys. Res. Sect. B Beam Interact. Mater. At.* **259**, 320–329 (2007).
7. Guerrero-Cruz, S. *et al.* Methanotrophs: Discoveries, Environmental Relevance, and a Perspective on Current and Future Applications. *Front. Microbiol.* **12**, 678057 (2021).
8. Trotsenko, Y. A. & Khmelenina, V. N. Biology of extremophilic and extremotolerant methanotrophs. *Arch. Microbiol.* **177**, 123–131 (2002).
9. Pol, A. *et al.* Methanotrophy below pH 1 by a new Verrucomicrobia species. *Nature* **450**, 874–878 (2007).
10. Khmelenina, V. N. Isolation and Characterization of Halotolerant Alkaliphilic Methanotrophic Bacteria from Tuva Soda Lakes. *Curr. Microbiol.* **35**, 257–261 (1997).
11. Kaluzhnaya, M. *et al.* Taxonomic Characterization of New Alkaliphilic and Alkalitolerant Methanotrophs from Soda Lakes of the Southeastern Transbaikal Region and description of Methylomicrobium buryatense sp.nov. *Syst. Appl. Microbiol.* **24**, 166–176 (2001).
12. Sorokin, D. Yu., Jones, B. E. & Gijs Kuenen, J. An obligate methylotrophic, methane-oxidizing Methylomicrobium species from a highly alkaline environment. *Extremophiles* **4**, 145–155 (2000).
13. Boden, R. *et al.* Complete Genome Sequence of the Aerobic Marine Methanotroph Methylomonas methanica MC09. *J. Bacteriol.* **193**, 7001–7002 (2011).
14. Bussmann, I. Methane and methane oxidation at two time series stations in Lake Constance. 96 data points PANGAEA https://doi.org/10.1594/PANGAEA.854446 (2015).
15. Pack, M. A. *et al.* Methane oxidation in the eastern tropical North Pacific Ocean water column. *J. Geophys. Res. Biogeosciences* **120**, 1078–1092 (2015).
16. Overholt, W. A. *et al.* Carbon fixation rates in groundwater similar to those in oligotrophic marine systems. *Nat. Geosci.* **15**, 561–567 (2022).
17. Ji, M. *et al.* Atmospheric trace gases support primary production in Antarctic desert surface soil. *Nature* **552**, 400–403 (2017).
18. Bastviken, D., Ejlertsson, J. & Tranvik, L. Measurement of Methane Oxidation in Lakes: A Comparison of Methods. *Environ. Sci. Technol.* **36**, 3354–3361 (2002).
19. Treude, T., Krüger, M., Boetius, A. & Jørgensen, B. B. Environmental control on anaerobic oxidation of methane in the gassy sediments of Eckernförde Bay (German Baltic). *Limnol. Oceanogr.* **50**, 1771–1786 (2005).
20. Bussmann, I., Hackbusch, S., Schaal, P. & Wichels, A. Methane distribution and oxidation around the Lena Delta in summer 2013. *Biogeosciences* **14**, 4985–5002 (2017).
21. Qin, Q. *et al.* Seasonality of water column methane oxidation and deoxygenation in a dynamic marine environment. *Geochim. Cosmochim. Acta* **336**, 219–230 (2022).
22. Castaldi, S. & Tedesco, D. Methane production and consumption in an active volcanic environment of Southern Italy. *Chemosphere* **58**, 131–139 (2005).
23. Pack, M. A. *et al.* A method for measuring methane oxidation rates using lowlevels of 14C-labeled methane and accelerator mass spectrometry. *Limnol. Oceanogr. Methods* **9**, 245–260 (2011).

24. Dittmar, T., Koch, B., Hertkorn, N. & Kattner, G. A simple and efficient method for the solid-phase extraction of dissolved organic matter (SPE-DOM) from seawater. *Limnol. Oceanogr. Methods* **6**, 230–235 (2008).
25. Benk, S. A. *et al.* Fueling Diversity in the Subsurface: Composition and Age of Dissolved Organic Matter in the Critical Zone. *Front. Earth Sci.* **7**, 296 (2019).
26. Heinze, B. M. *et al.* Old but not ancient: Rock-leached organic carbon drives groundwater microbiomes. *Sci. Total Environ.* **959**, 178212 (2025).
27. Parada, A. E., Needham, D. M. & Fuhrman, J. A. Every base matters: assessing small subunit rRNA primers for marine microbiomes with mock communities, time series and global field samples. *Environ. Microbiol.* **18**, 1403–1414 (2016).
28. Quince, C., Lanzen, A., Davenport, R. J. & Turnbaugh, P. J. Removing Noise From Pyrosequenced Amplicons. *BMC Bioinformatics* **12**, 38 (2011).
29. DeLong, E. F. Archaea in coastal marine environments. *Proc. Natl. Acad. Sci.* **89**, 5685–5689 (1992).
30. Vaksmaa, A., Jetten, M. S. M., Ettwig, K. F. & Lüke, C. McrA primers for the detection and quantification of the anaerobic archaeal methanotroph 'Candidatus Methanoperedens nitroreducens'. *Appl. Microbiol. Biotechnol.* **101**, 1631–1641 (2017).
31. Knapp, C. W., Fowle, D. A., Kulczycki, E., Roberts, J. A. & Graham, D. W. Methane monooxygenase gene expression mediated by methanobactin in the presence of mineral copper sources. *Proc. Natl. Acad. Sci.* **104**, 12040–12045 (2007).
32. Costello, A. M. & Lidstrom, M. E. Molecular Characterization of Functional and Phylogenetic Genes from Natural Populations of Methanotrophs in Lake Sediments. *Appl. Environ. Microbiol.* **65**, 5066–5074 (1999).
33. Gleeson, T., Befus, K. M., Jasechko, S., Luijendijk, E. & Cardenas, M. B. The global volume and distribution of modern groundwater. *Nat. Geosci.* **9**, 161–167 (2016).
34. Kotelnikova, S. V. & Pedersen, K. The Microbe-REX projekt: Microbial O 2 consumption in the Äspö tunnel. SKB PR HRL-98-11. (1998).
35. Bussmann, I., Fedorova, I. V., Juhls, B., Overduin, P. P. & Winkel, M. Dissolved methane concentrations and oxidation rates in the Lena Delta area, 2016-2018. 2 datasets Preprint at https://doi.org/10.1594/PANGAEA.920015 (2020).
36. Bussmann, I., Osudar, R. & Matousu, A. Methane concentrations and methane oxidation rates from Oct 2010 - March 2012 in the Elbe Estuary, from Hamburg to Cuxhaven, Germany. 644 data points PANGAEA https://doi.org/10.1594/PANGAEA.833923 (2014).
37. Griffiths, R. P., Caldwell, B. A., Cline, J. D., Broich, W. A. & Morita, R. Y. Field Observations of Methane Concentrations and Oxidation Rates in the Southeastern Bering Sea. *Appl. Environ. Microbiol.* **44**, 435–446 (1982).
38. Liu, Q. *et al.* Dynamics and Controls of Methane Oxidation in the Aerobic Waters of Eastern China Marginal Seas. *J. Geophys. Res. Oceans* **129**, e2023JC020280 (2024).
39. Mao, S.-H. *et al.* Aerobic oxidation of methane significantly reduces global diffusive methane emissions from shallow marine waters. *Nat. Commun.* **13**, 7309 (2022).
40. Rogener, M. K., Sipler, R. E., Hunter, K. S., Bronk, D. A. & Joye, S. B. Pelagic methane oxidation in the northern Chukchi Sea. *Limnol. Oceanogr.* **65**, 96–110 (2020).
41. Rogener, M. K. *et al.* Pelagic denitrification and methane oxidation in oxygen-depleted waters of the Louisiana shelf. *Biogeochemistry* **154**, 231–254 (2021).
42. Steinle, L. *et al.* Water column methanotrophy controlled by a rapid oceanographic switch. *Nat. Geosci.* **8**, 378–382 (2015).
43. Steinle, L. *et al.* Linked sediment and water-column methanotrophy at a man-made gas blowout in the North Sea: Implications for methane budgeting in seasonally stratified shallow seas. *Limnol. Oceanogr.* **61**, (2016).
44. Steinle, L. *et al.* Effects of low oxygen concentrations on aerobic methane oxidation in seasonally hypoxic coastal waters. *Biogeosciences* **14**, 1631–1645 (2017).
45. Uhlig, C., Kirkpatrick, J. B., D'Hondt, S. & Loose, B. Methane-oxidizing seawater microbial communities from an Arctic shelf. *Biogeosciences* **15**, 3311–3329 (2018).
46. Ward, B. B., Kilpatrick, K. A., Novelli, P. C. & Scranton, M. I. Methane oxidation and methane fluxes in the ocean surface layer and deep anoxic waters. *Nature* **327**, 226–229 (1987).

47. Leonte, M. *et al.* Rapid rates of aerobic methane oxidation at the feather edge of gas hydrate stability in the waters of Hudson Canyon, US Atlantic Margin. *Geochim. Cosmochim. Acta* **204**, 375–387 (2017).
48. Valentine, D. L., Blanton, D. C., Reeburgh, W. S. & Kastner, M. Water column methane oxidation adjacent to an area of active hydrate dissociation, Eel river Basin. *Geochim. Cosmochim. Acta* **65**, 2633–2640 (2001).
49. Aromokeye, D. A. *et al.* Rates and Microbial Players of Iron-Driven Anaerobic Oxidation of Methane in Methanic Marine Sediments. *Front. Microbiol.* **10**, 3041 (2020).
50. Grünke, S. *et al.* Niche differentiation among mat-forming, sulfide-oxidizing bacteria at cold seeps of the Nile Deep Sea Fan (Eastern Mediterranean Sea): Niche differentiation among sulfide oxidizers. *Geobiology* **9**, 330–348 (2011).
51. Iversen, N. & Jorgensen, B. B. Anaerobic methane oxidation rates at the sulfate-methane transition in marine sediments from Kattegat and Skagerrak (Denmark)1. *Limnol. Oceanogr.* **30**, 944–955 (1985).
52. Joye, S. B. *et al.* The anaerobic oxidation of methane and sulfate reduction in sediments from Gulf of Mexico cold seeps. *Chem. Geol.* **205**, 219–238 (2004).
53. Pimenov, N. *et al.* Microbial processes of carbon cycle as the base of food chain of Håkon Mosby Mud Volcano benthic community. *Geo-Mar. Lett.* **19**, 89–96 (1999).
54. Reeburgh, W. S. Anaerobic methane oxidation: Rate depth distributions in Skan Bay sediments. *Earth Planet. Sci. Lett.* **47**, 345–352 (1980).
55. Reeburgh, W. S. *et al.* Black Sea methane geochemistry. *Deep Sea Res. Part Oceanogr. Res. Pap.* **38**, S1189–S1210 (1991).
56. Ruff, S. E. *et al.* Microbial Communities of Deep-Sea Methane Seeps at Hikurangi Continental Margin (New Zealand). *PLoS ONE* **8**, e72627 (2013).
57. Savvichev, A. S. *et al.* Biogeochemical Activity of Methane-Related Microbial Communities in Bottom Sediments of Cold Seeps of the Laptev Sea. *Microorganisms* **11**, 250 (2023).
58. Savvichev, A. S. *et al.* Methane as an Organic Matter Source and the Trophic Basis of a Laptev Sea Cold Seep Microbial Community. *Geomicrobiol. J.* **35**, 411–423 (2018).
59. Savvichev, A. S. *et al.* Microbial Community Composition and Rates of the Methane Cycle Microbial Processes in the Upper Sediments of the Yamal Sector of the Southwestern Kara Sea. *Microbiology* **87**, 238–248 (2018).
60. Savvichev, A. S. *et al.* Microbial processes of the carbon and sulfur cycles in the Chukchi Sea. *Microbiology* **76**, 603–613 (2007).
61. Bussmann, I., Hackbusch, S. & Warnstedt, J. Methane concentrations and methane oxidation rates from Jan 2013 - Nov 2014 in the Elbe Estuary, from Hamburg to Helgoland, Germany. 1979 data points PANGAEA https://doi.org/10.1594/PANGAEA.897351 (2019).
62. Namsaraev, B. B. *et al.* Bacterial methane oxidation rates in waters and sediments of the Kara Sea and the Yenisey River estuary. 14 datasets Preprint at https://doi.org/10.1594/PANGAEA.746801 (1995).
63. Osudar, R. *et al.* Methane turnover and methanotrophic communities in arctic aquatic ecosystems of the Lena Delta, Northeast Siberia. *FEMS Microbiol. Ecol.* **92**, fiw116 (2016).
64. Abril, G. & Iversen, N. Methane dynamics in a shallow non-tidal estuary (Randers Fjord, Denmark). *Mar. Ecol. Prog. Ser.* **230**, 171–181 (2002).
65. Dzyuban, A. N. Methane and its transformation processes in water of some tributaries of the Rybinsk Reservoir. *Water Resour.* **38**, 615–620 (2011).
66. Matoušů, A. *et al.* Methane dynamics in a large river: a case study of the Elbe River. *Aquat. Sci.* **81**, 12 (2019).
67. Utsumi, M. *et al.* Oxidation of dissolved methane in a eutrophic, shallow lake: Lake Kasumigaura, Japan. *Limnol. Oceanogr.* **43**, 471–480 (1998).
68. Shelley, F., Abdullahi, F., Grey, J. & Trimmer, M. Microbial methane cycling in the bed of a chalk river: oxidation has the potential to match methanogenesis enhanced by warming. *Freshw. Biol.* **60**, 150–160 (2015).
69. Shelley, F., Grey, J. & Trimmer, M. Widespread methanotrophic primary production in lowland chalk rivers. *Proc. R. Soc. B Biol. Sci.* **281**, 20132854 (2014).
70. Shen, L., Ouyang, L., Zhu, Y. & Trimmer, M. Active pathways of anaerobic methane oxidation across contrasting riverbeds. *ISME J.* **13**, 752–766 (2019).

71. Bussmann, I. & Wessels, M. Hydrography of water column, methane, and methane oxidation rates at two time series stations in Lake Constance. 9 datasets Preprint at https://doi.org/10.1594/PANGAEA.854468 (2015).
72. Carini, S., Bano, N., LeCleir, G. & Joye, S. B. Aerobic methane oxidation and methanotroph community composition during seasonal stratification in Mono Lake, California (USA). *Environ. Microbiol.* **7**, 1127–1138 (2005).
73. Donis, D. *et al.* Full-scale evaluation of methane production under oxic conditions in a mesotrophic lake. *Nat. Commun.* **8**, 1661 (2017).
74. Dumestre, J. F. *et al.* Influence of Light Intensity on Methanotrophic Bacterial Activity in Petit Saut Reservoir, French Guiana. *Appl. Environ. Microbiol.* **65**, 534–539 (1999).
75. Oswald, K. *et al.* Light-Dependent Aerobic Methane Oxidation Reduces Methane Emissions from Seasonally Stratified Lakes. *PLOS ONE* **10**, e0132574 (2015).
76. Saxton, M. A. *et al.* Biogeochemical and 16S rRNA gene sequence evidence supports a novel mode of anaerobic methanotrophy in permanently ice-covered Lake Fryxell, Antarctica. *Limnol. Oceanogr.* **61**, (2016).
77. Schorn, S. *et al.* Persistent activity of aerobic methane-oxidizing bacteria in anoxic lake waters due to metabolic versatility. *Nat. Commun.* **15**, 5293 (2024).
78. Van Grinsven, S. *et al.* Methane oxidation in anoxic lake water stimulated by nitrate and sulfate addition. *Environ. Microbiol.* **22**, 766–782 (2020).
79. Deutzmann, J. S. & Schink, B. Anaerobic Oxidation of Methane in Sediments of Lake Constance, an Oligotrophic Freshwater Lake. *Appl. Environ. Microbiol.* **77**, 4429–4436 (2011).
80. Norði, K. À., Thamdrup, B. & Schubert, C. J. Anaerobic oxidation of methane in an iron-rich Danish freshwater lake sediment. *Limnol. Oceanogr.* **58**, 546–554 (2013).
81. Pimenov, N. V., Kalmychkov, G. V., Veryasov, M. B., Sigalevich, P. A. & Zemskaya, T. I. Microbial oxidation of methane in the sediments of central and southern Baikal. *Microbiology* **83**, 773–781 (2014).
82. Takeuchi, M. *et al.* A distinct freshwater-adapted subgroup of ANME-1 dominates active archaeal communities in terrestrial subsurfaces in Japan. *Environ. Microbiol.* **13**, 3206–3218 (2011).
83. Vigderovich, H. *et al.* Long-term incubations provide insight into the mechanisms of anaerobic oxidation of methane in methanogenic lake sediments. *Biogeosciences* **19**, 2313–2331 (2022).
84. Chen, L. *et al.* Anaerobic methane oxidation linked to Fe(III) reduction in a CANDIDATUS METHANOPEREDENS - enriched consortium from the cold Zoige wetland at Tibetan Plateau. *Environ. Microbiol.* **24**, 614–625 (2022).
85. Krause, S. J. E. & Treude, T. Deciphering cryptic methane cycling: Coupling of methylotrophic methanogenesis and anaerobic oxidation of methane in hypersaline coastal wetland sediment. *Geochim. Cosmochim. Acta* **302**, 160–174 (2021).
86. Segarra, K. E. A., Comerford, C., Slaughter, J. & Joye, S. B. Impact of electron acceptor availability on the anaerobic oxidation of methane in coastal freshwater and brackish wetland sediments. *Geochim. Cosmochim. Acta* **115**, 15–30 (2013).
87. Segarra, K. E. A. *et al.* High rates of anaerobic methane oxidation in freshwater wetlands reduce potential atmospheric methane emissions. *Nat. Commun.* **6**, 7477 (2015).
88. Shi, Y. *et al.* Using 13C isotopes to explore denitrification-dependent anaerobic methane oxidation in a paddy-peatland. *Sci. Rep.* **7**, 40848 (2017).
89. Valenzuela, E. I. *et al.* Anaerobic Methane Oxidation Driven by Microbial Reduction of Natural Organic Matter in a Tropical Wetland. *Appl. Environ. Microbiol.* **83**, e00645-17 (2017).
90. Wang, W. *et al.* Soil Methane Production, Anaerobic and Aerobic Oxidation in Porewater of Wetland Soils of the Minjiang River Estuarine, China. *Wetlands* **38**, 627–640 (2018).
91. Wang, J. *et al.* Spatial-Temporal Pattern of Sulfate-Dependent Anaerobic Methane Oxidation in an Intertidal Zone of the East China Sea. *Appl. Environ. Microbiol.* **85**, e02638-18 (2019).
92. Wang, Z., Li, J., Xu, X., Li, K. & Chen, Q. Denitrifying anaerobic methane oxidation and mechanisms influencing it in Yellow River Delta coastal wetland soil, China. *Chemosphere* **298**, 134345 (2022).
93. Wang, Z. *et al.* Soil nitrogen substances and denitrifying communities regulate the anaerobic oxidation of methane in wetlands of Yellow River Delta, China. *Sci. Total Environ.* **857**, 159439 (2023).
94. Xie, F. *et al.* Niche differentiation of denitrifying anaerobic methane oxidizing bacteria and archaea leads to effective methane filtration in a Tibetan alpine wetland. *Environ. Int.* **140**, 105764 (2020).

95. Zhang, M. *et al.* Molecular and stable isotopic evidence for the occurrence of nitrite-dependent anaerobic methane-oxidizing bacteria in the mangrove sediment of Zhangjiang Estuary, China. *Appl. Microbiol. Biotechnol.* **102**, 2441–2454 (2018).
96. Zhang, Y., Zhang, X., Wang, F., Xia, W. & Jia, Z. Exogenous nitrogen addition inhibits sulfate-mediated anaerobic oxidation of methane in estuarine coastal sediments. *Ecol. Eng.* **158**, 106021 (2020).
97. Zhang, M. *et al.* Finding the pieces for the anaerobic methane oxidation jigsaw puzzle in mangrove wetlands. *Int. Biodeterior. Biodegrad.* **168**, 105375 (2022).
98. Zhao, Y., Jiang, H., Wang, X., Liu, C. & Yang, Y. Quinolone antibiotics enhance denitrifying anaerobic methane oxidation in Wetland sediments: Counterintuitive results. *Environ. Pollut.* **305**, 119300 (2022).
99. Zhu, B. *et al.* Anaerobic Oxidization of Methane in a Minerotrophic Peatland: Enrichment of Nitrite-Dependent Methane-Oxidizing Bacteria. *Appl. Environ. Microbiol.* **78**, 8657–8665 (2012).
100. Magnabosco, C. *et al.* Comparisons of the composition and biogeographic distribution of the bacterial communities occupying South African thermal springs with those inhabiting deep subsurface fracture water. *Front. Microbiol.* **5**, (2014).
101. Kulongoski, J. T. & McMahon, P. B. Methane emissions from groundwater pumping in the USA. *Npj Clim. Atmospheric Sci.* **2**, 11 (2019).
102. Moritz, A. *et al.* Methane Baseline Concentrations and Sources in Shallow Aquifers from the Shale Gas-Prone Region of the St. Lawrence Lowlands (Quebec, Canada). *Environ. Sci. Technol.* **49**, 4765–4771 (2015).
103. Smedley, P. L. *et al.* Monitoring of methane in groundwater from the Vale of Pickering, UK: Temporal variability and source discrimination. *Chem. Geol.* **636**, 121640 (2023).
104. GrÃ¼ndger, F. *et al.* Microbial methane formation in deep aquifers of a coal-bearing sedimentary basin, Germany. *Front. Microbiol.* **6**, (2015).
105. Glodowska, M. *et al.* Arsenic mobilization by anaerobic iron-dependent methane oxidation. *Commun. Earth Environ.* **1**, 42 (2020).
106. Morgenstern, U., Moreau, M., Coble, M. A., Johnson, K. & Townsend, D. B. Groundwater and surface water conceptual flow from environmental tracer signatures in the Pukekohe and Bombay area. (2023) doi:10.21420/VNBF-3X96.
107. Tian, H. *et al.* Identification of methane cycling pathways in Quaternary alluvial-lacustrine aquifers using multiple isotope and microbial indicators. *Water Res.* **250**, 121027 (2024).
108. Schout, G., Griffioen, J., Hartog, N., Eggenkamp, H. G. M. & Cirkel, D. G. Methane occurrence and origin in Dutch groundwater: from shallow aquifers to deep reservoirs. *Neth. J. Geosci.* **103**, e24 (2024).
109. Atkins, M. L., Santos, I. R. & Maher, D. T. Groundwater methane in a potential coal seam gas extraction region. *J. Hydrol. Reg. Stud.* **4**, 452–471 (2015).
110. Magnabosco, C. *et al.* Fluctuations in populations of subsurface methane oxidizers in coordination with changes in electron acceptor availability. *FEMS Microbiol. Ecol.* **94**, (2018).
111. Hallbeck, L. & Pedersen, K. Characterization of microbial processes in deep aquifers of the Fennoscandian Shield. *Appl. Geochem.* **23**, 1796–1819 (2008).
112. Lin, L. *et al.* Radiolytic H_2 in continental crust: Nuclear power for deep subsurface microbial communities. *Geochem. Geophys. Geosystems* **6**, 2004GC000907 (2005).
113. Pedersen, K. Microbial life in deep granitic rock. *FEMS Microbiol. Rev.* **20**, 399–414 (1997).
114. Pedersen, K. *et al.* Numbers, biomass and cultivable diversity of microbial populations relate to depth and borehole-specific conditions in groundwater from depths of 4–450 m in Olkiluoto, Finland. *ISME J.* **2**, 760–775 (2008).
115. Ward, J. A. *et al.* Microbial hydrocarbon gases in the Witwatersrand Basin, South Africa: Implications for the deep biosphere. *Geochim. Cosmochim. Acta* **68**, 3239–3250 (2004).
116. Iwatsuki, T., Furue, R., Mie, H., Ioka, S. & Mizuno, T. Hydrochemical baseline condition of groundwater at the Mizunami underground research laboratory (MIU). *Appl. Geochem.* **20**, 2283–2302 (2005).
117. Haveman, S. A., Pedersen, K. & Ruotsalainen, P. Distribution and Metabolic Diversity of Microorganisms in Deep Igneous Rock Aquifers of Finland. *Geomicrobiol. J.* **16**, 277–294 (1999).
118. Hohne, D., De Lange, F., Esterhuyse, S. & Sherwood Lollar, B. Case study: methane gas in a groundwater system located in a dolerite ring structure in the Karoo Basin; South Africa. *South Afr. J. Geol.* **122**, 357–368 (2019).
